# Novel Naproxen Salts with Increased Skin Permeability

**DOI:** 10.3390/pharmaceutics13122110

**Published:** 2021-12-07

**Authors:** Ewelina Świątek, Paula Ossowicz-Rupniewska, Ewa Janus, Anna Nowak, Peter Sobolewski, Wiktoria Duchnik, Łukasz Kucharski, Adam Klimowicz

**Affiliations:** 1 Department of Chemical Organic Technology and Polymeric Materials, Faculty of Chemical Technology and Engineering, West Pomeranian University of Technology in Szczecin, Piastów Ave. 42, PL-71065 Szczecin, Poland; ewelinaswiatek94@gmail.com (E.Ś.); ejanus@zut.edu.pl (E.J.); 2 Department of Cosmetic and Pharmaceutical Chemistry, Pomeranian Medical University in Szczecin, Powstańców Wielkopolskich Ave. 72, PL-70111 Szczecin, Poland; anowak@pum.edu.pl (A.N.); wiktoria.duchnik@pum.edu.pl (W.D.); lukasz.kucharski@pum.edu.pl (Ł.K.); adklim@pum.edu.pl (A.K.); 3 Department of Polymer and Biomaterials Science, Faculty of Chemical Technology and Engineering, West Pomeranian University of Technology in Szczecin, Piastów Ave. 45, PL-70311 Szczecin, Poland; psobolewski@zut.edu.pl

**Keywords:** amino acid, naproxen, ionic liquids, nonsteroidal anti-inflammatory drug, transdermal drug delivery, skin barrier

## Abstract

The paper presents the synthesis, full identification, and characterization of new salts-L-proline alkyl ester naproxenates [ProOR][NAP], where R was a chain from ethyl to butyl (including isopropyl). All obtained compounds were characterized by Nuclear Magnetic Resonance (NMR), Fourier transform infrared spectroscopy (FTIR), X-ray powder diffractometry (XRD), and in vitro dissolution studies. The specific rotation, phase transition temperatures (melting point), and thermal stability were also determined. In addition, their lipophilicity, permeability, and accumulation in pigskin were determined. Finally, toxicity against mouse L929 fibroblast cells was tested. The obtained naproxen derivatives showed improved solubility and higher absorption of drug molecules by biological membranes. Their lipophilicity was lower and increased with the increase in the alkyl chain of the ester. The derivative with isopropyl ester had the best permeability through pigskin. The use of L-proline isopropyl ester naproxenate increased the permeation of naproxen through the skin almost four-fold. It was also shown that the increase in permeability is not associated with additional risk: all compounds had a similar effect on cell viability as the parent naproxen.

## 1. Introduction

Bioavailability is a term that defines the speed and degree of absorption of an active substance, which determines the therapeutic effect’s effectiveness. A wide variety of factors determine whether the bioavailability of a particular active ingredient will be high or low. These factors are related to the properties of the drug substance, its function, and its physiological conditions. The physicochemical properties of a substance can be regarded as playing a pivotal role in predicting its possible use as a drug [1,2,3,4]. These properties determine both the speed of dissolution and the possibility and speed of permeation through biological membranes. Together, these aspects yield the probability of reaching a concentration that is considered therapeutic at the site of action [5].

There are many known methods to increase nonsteroidal anti-inflammatory drug solubility and bioavailability [6,7,8,9,10,11,12,13]. In the present paper, we focused on chemically modifying an active compound. We chose naproxen as the active compound. Naproxen is a popular nonsteroidal anti-inflammatory drug. However, its poor solubility in water (0.051 mg cm^−3^ [14]) has limited its bioavailability. As a result, several studies have focused on obtaining naproxen prodrugs with potentially improved bioavailability. For example, Aboul-Fadl et al., obtained naproxen amides with amino acid esters. Prodrugs derived from naproxen and ethyl glycinate or L-valine ethyl ester showed promising results in the release of naproxen. Furthermore, modifications of naproxen had colon tumor cell growth inhibitory activity through cyclooxygenase (COX)-independent mechanism. Their results showed a lack of COX inhibitory activity and indicated a superior gastrointestinal tract safety profile for the synthesized compounds compared to the parent drug naproxen [15].

Another promising strategy is using co-amorphous salts of amino acids with naproxen to create physically stable amorphous systems with increased water solubility. Thus far, tryptophan, arginine, and proline have been studied; the obtained co-amorphic mixtures were tested for stability and increased the dissolution rate of naproxen [16,17]. Ternary systems of naproxen with hydroxypropyl-beta-cyclodextrin and amino acids are also known, which have improved drug solubility [18,19]. Finally, obtaining an ionic liquid based on an active substance (active pharmaceutical ingredient-based ionic liquids (API-ILs)) is also a very interesting approach. Ionic liquids are a promising solution to many pharmaceutical problems, particularly the low solubility and hence the bioavailability of active pharmaceutical ingredients and the presence of polymorphs that limit the effectiveness of essential commercial used drugs [20]. These compounds consist quasi-exclusively of ions and are characterized by a low melting point (below 100 °C) and many desirable properties, i.e., negligible vapor pressure, high stability (thermal, chemical, and also electrochemical), widely tunable properties with regard to polarity, hydrophobicity, and solvent miscibility [21,22]. The properties of these compounds can be suitably designed by selecting the ions of the ionic liquid. There are many known uses of ionic liquids in pharmacy. For example, choline-based ionic liquids with an anion derived from ketoprofen and naproxen with better solubility and better ability to interact with biological membranes are known. Naproxen with the choline cation showed four times greater solubility compared to naproxen sodium. Increasing the solubility may positively affect the bioavailability of the drug and thus improve its absorption. These compounds also showed promising anti-inflammatory properties [23].

Here, we aimed to develop new API-ILs with biocompatible cations, such as those derived from proline. L-Proline is the only one of the twenty DNA-encoded amino acids with a secondary amino group alpha to the carboxyl group. L-proline, after transformation into hydroxyl-proline, is part of collagen and constitutes 1/3 of its proteins. Therefore, it is necessary for the proper functioning of the locomotor system, supporting the functioning of joints, tendons, and cartilage. As a result, L-proline supplementation can be crucial in the period of injury, rehabilitation, and intense training. Likewise, as a component of collagen, it contributes to the elasticity of the artery walls, which is essential for the proper function of the circulatory system. It also helps maintain and strengthen heart muscles. It is of great importance in healing damaged tissues, such as wounds, burns, or connective tissue disorders. It is responsible for the youthful appearance of the skin as well as the excellent condition of hair and nails. Proline is used in sports supplementation, and it helps build lean muscle mass and accelerates regeneration after training [24]. In addition, L-proline and its metabolite (P5C) are known to regulate gene expression and cell signaling pathways that are essential for health [25].

As a result, L-proline derivatives seemed to be a natural fit in our search for new cations to improve solubility and permeation of naproxen, particularly in the context of topical applications, such as those following muscle or joint injury. Therefore, we explored a new combination of acid–base complexes using protonated alkyl esters of L-proline as the cation. Toward this aim, we developed a new solvent-free synthesis method. We present the synthesis, full identification, and characterization of new salts, L-proline alkyl ester naproxenates [ProOR][NAP], where R was a chain from ethyl to butyl (including isopropyl).

## 2. Materials and Methods

### 2.1. Materials

All reagents were commercially available materials and were used without further purification. (+)-(*S*)-2-(6-Methoxynaphthalen-2-yl)propanoic acid (≥98%) was provided from AmBeed (Arlington Heights, IL, USA). L-proline (≥98.5%) was obtained from Carl Roth (Karlsruhe, Germany). Trimethylsilyl chloride (≥99%) (TMSCl) was purchased from Sigma-Aldrich (Steinheim am Albuch, Germany). Ammonium hydroxide solution 25% (NH_3_∙H_2_O) was of analytical grade obtained from StanLab (Lublin, Poland). Methanol (MeOH), ethanol (EtOH), propan-2-ol (i-PrOH), propan-1-ol (PrOH), butan-1-ol (BuOH), dimethyl sulfoxide, chloroform, ethyl acetate, diethyl ether, toluene, and n-hexane of high purity were purchased from Chempur (Gliwice, Poland). Deuterated chloroform (CDCl_3_) (99.8%) (+0.03% TMSCl) was provided by Eurisotop (Cheshire, England). Acetonitrile was provided by J.T. Baker (Radnor, Pennsylvania, USA). PBS (pH 7.4), potassium dihydrogen phosphate (p.a.) was purchased from Merck (Darmstadt, Germany). For cell culture studies, murine fibroblast cell line, L929, and all cell culture reagents were purchased from Sigma Aldrich (Poznań, Poland), while all sterile, single-use cell culture plasticware was purchased from VWR (Gdańsk, Poland).

### 2.2. Skin

In the experiment, porcine skin was used due to its similar permeability and thickness to human skin [26,27]. The local slaughterhouse provided the skin. The skin thickness of 0.5 mm was excised by a dermatome, wrapped into aluminum foil, and stored at −20 °C until use. Under these conditions, the skin was used no later than 3 months. This freezing time ensured the stability of the skin barrier properties [28]. Before the experiment, the skin was thawed at room temperature for about 30 min and then was soaked in a PBS (pH 7.4) solution for 15 min to hydrate it [29,30]. The skin pieces were placed between the donor and acceptor chamber of Franz diffusion cells, and then, the integrity of the skin was checked by measuring its impedance. Skin impedance was measured using an LCR meter 4080 (Conrad Electronic, Germany) [31]. For all experiments, only skin samples with impedance > 3 kΩ were used—these values are similar to the electrical resistance for human skin [32].

### 2.3. Synthesis of the L-Proline Alkyl Ester Naproxenate [ProOR][NAP]

**Scheme 1:**
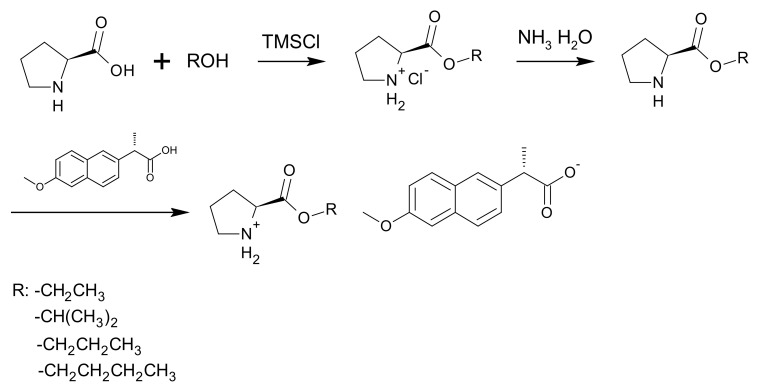
General synthetic path for [ProOR][NAP].

First, L-proline alkyl esters (L-ProOR) necessary for the reaction were obtained according to the known and previously described method consisting of a two-step reaction (1-step preparation of the hydrochloride of the alkyl ester and 2-step-neutralization of the hydrochloride) [33,34]. Then, naproxen derivatives were synthesized using a newly developed solvent-free method (Scheme 1). An equimolar amount of naproxen was added to each L-ProOR and mixed. The mixture was stirred thoroughly at room temperature for 1 h. Then, the obtained naproxen derivatives were dried in a vacuum oven at 60 °C, for 24 h. All syntheses were performed at least three times on a scale of 0.5–2 g of product.

The purity and identity of obtained compounds were confirmed by NMR, FT-IR, and elemental analysis methods.

### 2.4. General Analytical Methods

#### 2.4.1. Identification and Properties of [ProOR][NAP]

The structure and purity of the obtained compounds were confirmed by spectroscopic methods (^1^H NMR, ^13^C NMR, ATR-FTIR, UV-Vis) and elemental analysis.

The H^1^ and C^13^ nuclear magnetic resonance (NMR) spectra were recorded with a BRUKER DPX-400 spectrometer (Billerica, MA, USA) at 400 MHz (^1^H) and 100 MHz (^13^C) in CDCl_3_ as a solvent. The chemical shifts (δ, ppm) are given relative to TMS used as the internal standard.

The attenuated total reflectance Fourier transform infrared (ATR-FTIR) spectra data were collected on Thermo Scientific Nicolet 380 spectrometer (Waltham, MA, USA) equipped with an ATR diamond plate. The spectra were recorded in transmission mode in the range of 4000–400 cm^−1^ at the resolution of 4 cm^−1^.

The ultraviolet-visible (UV-Vis) spectra were recorded on a Spectroquant^®^ Pharo 300 Spectrophotometer from Merck (Darmstadt, Germany). The solutions were prepared in absolute ethanol of concentration range 10^−4^–10^−5^ M. The measurements were performed in a 10 mm quartz cell in the wavelength range of 190–400 nm with the accuracy of ±1 nm.

The determination of elemental composition was performed using Thermo Scientific™ FLASH 2000 CHNS/O Analyzer (Waltham, MA, USA). The individual elements were detected by a thermal conductivity detector (TCD). The reactor temperature is 1060 °C for oxygen analysis and 950 °C for the rest of the elements. Three replications were performed for each compound. The samples were prepared in the tin (CHNS analysis) or silver (O analysis) crucibles and were weighed with an accuracy of ± 0.000001 g. The content of individual elements was determined using the calibration curve method. 2,5-Bis(5-tert-butyl-2-benzo-oxazol-2-yl)thiophene, L-cysteine, L-methionine, and sulfanilamide were used as standards in CHNS-mode, and acetanilide and benzoic acid were used for calibration in O-mode, respectively.

To verify the crystallinity of the synthesized materials, X-ray powder diffraction analysis (XRD) was recorded by PANalytical Empyrean X-ray diffractometer (Malvern Panalytical, Malvern, UK) using CuKα radiation (λ = 1.54056 Å). The phase composition of unmodified S-(+)-naproxen was determined using Panalytical X’Pert HighScore software with ICDD PDF4+ database.

The thermal stability, phase transition temperatures, specific rotation, solubility in water, PBS (pH 7.4), typical organic solvents, and the octanol–water partition coefficient were also determined for all compounds.

The thermogravimetric (TG) analysis was determined on a Netzsch Proteus Thermal Analysis TG 209 F1 Libra apparatus (Selb, Germany). The study was performed under an oxidizing atmosphere, the nitrogen flow was 10 cm^3^ min^−1^, and the airflow was 25 cm^3^ min^−1^. The temperature range was 25–1000 °C. The tests were carried out using alumina crucibles (Al_2_O_3_), and the sample weight was in the field of 5–10 mg.

Phase transition temperatures were recorded by differential scanning calorimetry (DSC) on TA Instruments, model Q100 DSC (New Castle, DE, USA). The sample was loaded on an aluminum pan with a pierced lid. The analysis was carried out in a nitrogen atmosphere. The sample was first cooled from 20 to 0 °C (without the collection of data) and then was heated from 0 °C to specified temperature; then, it was again cooled to 0 °C and heated to the specified temperature. The rate of heating/cooling/heating was 10 °C min^−1^. The specified temperature was the individual temperature for each compound, and it was at least 10 °C lower than the onset decomposition temperature, as determined from TG analysis. Indium and mercury were used as standards to calibrate the temperature. Heat calibration used indium.

The melting point was measured using an MPA100 Melting Point Apparatus with an automated melting point system from SRS—Stanford Research Systems (Sunnyvale, CA, USA). Measurements were carried out within the temperature range of 25 to 400 °C. The heating rate was 2 °C min^−1^, and measurement accuracy was 0.3 °C.

The specific rotation [α]_D_^20^ was tested using the Autopol IV automatic polarimeter from Rudolph Research Analytical (Hackettstown, NJ, USA) at the temperature of 20 °C and the wavelength of 589 nm. The measurements were collected for ethanol solutions with a concentration of 0.01 g cm^−3^.

The determination of pKa for L-proline alkyl esters was made by potentiometric acid–base titration using a pH meter CP-505 (Elmetron, Zabrze, Poland) equipped with an electrode EPS-1 (Elmetron, Zabrze, Poland). First, to a solution of 0.65 mM of the proper L-proline alkyl ester hydrochloride, HCl aqueous solution was added in an amount that allowed reaching pH below 4.0. Then, an acid–base titration was carried out by adding 5 mM NaOH solution dropwise. At least three replicates were made for each proline ester hydrochloride.

The pKs values from obtained salts were calculated from the equation:

pKs = pKA + pKB − pKw.

The solubility of the naproxen and its derivatives in water and phosphate-buffered saline (PBS (pH 7.4)) buffer solution was performed by assessing the concentration of saturated solutions. Then, the obtained saturated solutions were diluted 100 times and analyzed. The analysis was made using high-performance liquid chromatography (HPLC) with a DAD/FLD detector-SHIMADZU Nexera-i LC-2040C 3D High Plus liquid chromatograph (Kyoto, Japan). A mixture of 50% acetonitrile and 50% of water was used as the mobile phase. The flow rate was 1 cm^3^ min^−1^. The column temperature was 30 °C. A Kinetex^®^ 2.6 µm F5 100 Å column with dimensions 150 × 4.6 mm was used. The injection volume for these samples was 50 mm^3^. Each measurement was performed in triplicate, and the results were averaged.

The solubility in typical polar and nonpolar organic solvents was evaluated by modified Vogel’s method at a temperature of 25 °C [35]. By this method, the compound was classified as soluble, partially soluble, and insoluble. The compound was recognized as soluble when more than 100 mg of the compound dissolved in 1 cm^3^, partly soluble when 33–100 mg of compound dissolved in 1 cm^3^, and practically insoluble when less than 33 mg of the compound dissolved in 1 cm^3^. Dimethyl sulfoxide, ethanol, chloroform, ethyl acetate, diethyl ether, toluene, and n-hexane were used as solvents.

The n-octanol/water partition coefficient was assessed using the shake flask method. Next, the concentration of the substance in the aqueous layer was determined by high-performance liquid chromatography (HPLC), using a SHIMADZU Nexera-i LC-2040C 3D High Plus liquid chromatograph with a DAD/FLD detector. HPLC parameters were the same as described above for the dissolution test.

The microscopic examinations were performed on the optical microscope MBL 180T (Delta Optical, Mińsk Mazowiecki, Poland) with the MC500-W3 5MP camera (CMOS sensor, USB connector).

#### 2.4.2. Methodology of Skin Permeation and Accumulation

Skin permeation experiments were performed by using Franz diffusion cells (SES GmbH Analyse Systeme, Germany). The receptor fluid was PBS (pH 7.4). The diffusion area was 1 cm^2^. The receptor chamber (8 cm^3^) was maintained at a constant temperature of 32 ± 0.5 °C via thermostat (VEB MLW Prüfgeräte-Werk type of 3280). The content of the acceptor chamber was stirred with a magnetic stirring bar at the same speed for all cells. The donor chamber was filled with 1 cm^3^ of the saturated solutions of tested compounds in PBS (pH 7.4). The experiment was carried out over 24 h. In order to prevent evaporation of the acceptor fluid, the donor chamber containing the acceptor fluid was covered with a plastic cap. Samples were collected after 0.5 h, 1 h, 2 h, 3 h, 4 h, 5 h, 8 h, and 24 h of stirring. At each time point, samples of the acceptor fluid (0.3 cm^3^) were withdrawn and refilled with fresh buffer at the same pH. The concentration of naproxen and the obtained derivatives in the acceptor phase were measured using HPLC. After the end of the experiment, the donor chamber was disconnected from the Franz diffusion cell, and each skin sample was removed and carefully rinsed in PBS (pH 7.4) solution. Then, the skin samples were dried at room temperature, weighed and cut by diffusion area (1 cm^2^), and minced using scissors. Next, the skin samples were placed in 2 cm^3^ methanol and were incubated for 24 h at 4 °C. After this time, skin samples were homogenized for 3 min using a homogenizer (IKA^®^T18 digital ULTRA TURRAX). The homogenate was centrifuged at 3500 rpm for 5 min. Finally, the supernatant was collected and analyzed using HPLC. The cumulative mass (μg cm^−2^) was calculated based on this concentration.

The permeation parameters–fluxes of naproxen and its derivatives through the skin (J_SS_), the diffusion coefficient (K_P_), and the time required to reach steady-state permeation (lag time–L_T_) were obtained from typically J-shaped profiles by using the following equation:

A = J_ss_(t − L_T_)

where A is the cumulative amount (in μg IBU·cm^−2^) of IBU and its salts permeating into the receptor compartment, Jss is the steady-state flux (in μg∙cm^−2^∙h^−1^), t is the time (h), and L_T_ is the lag time (h).

The steady-state flux (in μg cm^−2^ h^−1^) was estimated from the slope of the linear portion of the plot of cumulative mass in the acceptor phase over time. The lag time (L_T_) was determined from the x-intercept of the linear part of the plot of cumulative permeation mass in the acceptor phase over time and was used to calculate the diffusion coefficient (K_P_) as follows:

K_P_ = Jss/C

where C is the concentration in the donor phase.

#### 2.4.3. HPLC

The liquid chromatography system (Knauer, Berlin, Germany) used in skin permeation experiments to determine the concentration of compounds in acceptor fluid and accumulation in the skin consisted of the following units: a Smartline model 1050 pump, model 2600 UV detector, and Smartline model 3950 autosampler. ClarityChrom 2009 software was applied for instrument control and data acquisition and processing. The 125 × 4 mm chromatographic column filled with Hyperisil ODS (C18), particle size 5 µm, was used. The detector was operated at 270 nm. The mobile phase was 0.02 M potassium dihydrogen phosphate–acetonitrile (60/40 *v*/*v*) with a flow rate of 1 cm^3^ min^−1^. The column temperature was set at 25 °C, and the injection volume was 20 μL.

#### 2.4.4. Cytotoxicity Experiments

The obtained compounds were screened for cytotoxicity in vitro based on ISO-10993-5 [36] and the NCI60 [37] dose–response methodology. Murine fibroblast cell line, L929, (passages 7–25) was maintained in Dulbecco’s Modified Eagle Medium (DMEM) supplemented with 10% fetal bovine serum (FBS), 2 mM L-glutamine, 100 U cm^−3^ penicillin, and 100 μg cm^−3^ streptomycin. For the cytotoxicity study, L929 cells were plated in 96-well plates, 10,000 cells per well, and incubated for 24 h to permit for attachment and spreading. At this time, the media was aspirated and replaced with media containing the NAP derivatives or naproxen, as a control. All compounds were dissolved in pre-warmed cell culture media directly prior to the experiment. For the NAP derivatives, the highest concentration used was approximately 2 mM, and eight 3-fold serial dilutions were performed. Thus, the tested concentrations spanned approximately 0.3–2000 µM. For naproxen, due to reduced solubility, the maximum concentration prepared was approximately 1.5 mM, and the eight 3-fold serial dilutions yielded a span of concentrations of 0.2–1500 µM. For each concentration, 6 technical replicates were performed. After 24 h of culture with the tested compounds, cell viability was assessed using light microscopy (Delta Optical IB-100) and using the resazurin viability assay [38]. Briefly, 20 mm^3^ of resazurin stock (0.15 mg cm^−3^ in PBS (pH 7.4)) was added to each well, the plates were incubated for 4 h at 37 °C in a cell culture incubator, and fluorescence was measured using a BioTek Synergy HTX multifunctional plate reader (Ex: 540 nm, Em: 590 nm). To ensure that the tested compounds did not interfere with the viability assay, samples of media with the highest concentration of each compound were tested in the same fashion. Fluorescence data were blank-subtracted and normalized to sham wells where the replacement media did not contain any compound.

#### 2.4.5. Statistical Analysis

Results are presented as the mean ± standard deviation (SD). A one-way analysis of variance (ANOVA) was used. In the case of the cumulative mass, the significance of differences between individual groups was evaluated with Tukey’s test (α < 0.05). A cluster analysis was carried out to determine similarities between all compounds tested, taking into account all time points. On this basis, presented groups of compounds with a similar permeation. Significant differences in the cumulative mass between all analyzed compounds, taking into account all time points during the entire 24 h permeation, were estimated by the Mann–Whitney test, where each derivative was compared to the control. Statistical calculations were done using Statistica 13 PL software (StatSoft, Kraków, Polska).

## 3. Results and Discussion

### 3.1. Synthesis and Identification of [ProOR][NAP]

The synthesis of L-proline alkyl ester of naproxen was performed using a modified three-step method described previously [34]. The synthesis and properties of the L-valine and L-leucine alkyl (ethyl, propyl, isopropyl, and butyl) ester salt have been described in previous work [34]. This study uses new salts, L-proline alkyl ester salts, obtained by reacting an alkyl ester with naproxen without using a solvent. As is known, limiting the use of volatile organic solvents is very beneficial both in economic and ecological terms. The reaction gave products in high yields of 97–99% (Table 1). A high reproducibility of syntheses was obtained. The length of the alkyl chain (R) ester group was from C2 to C4, and the C3 group was used as a straight-chain n-propyl and branched-chain isopropyl group.

The resulting compounds were identified by ^1^H and ^13^C-NMR, FTIR, and elemental analysis. All compounds are solids under ambient conditions. ^1^H and ^13^C NMR spectra, ATR-FTIR spectra, and TG and DSC curves for [ProOR][NAP] are available in Figures S1–S20 (Supplementary Materials).

The identity and purity of the obtained compounds were confirmed. In this research, similar to in our previous study [31,39], we showed that the elongation of the alkyl chain in the amino acid molecule influences the physicochemical properties of these compounds, such as their stability, lipophilicity, melting point, thermal stability, dissolution in water, buffer solutions, and permeability to the skin.

[ProOEt][NAP]–L-proline ethyl ester naproxenate.

L-proline ethyl ester naproxenate was obtained as a white solid in a 98% yield.


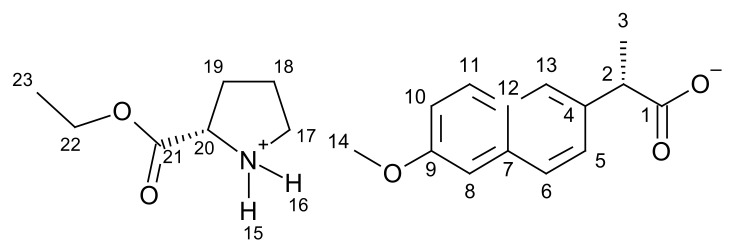


**^1^H NMR** (400 MHz, CDCl_3_) δ in ppm: 8.70 (s, 2H, H15, H16); 7.67–7.63 (s+d, 3H, H6, H8, H11); 7.45–7.43 (dd,1H, H10); 7.10–7.07 (s+d, 2H, H5, H13); 4.17–4.11 (q, 2H, H22); 4.02–3.99 (q, 1H, H20); 3.89 (s, 3H, H14); 3.79–3.73 (q, 1H, H2); 3.02 (t, J_18,17_ = 7.1 Hz, 2H, H17); 2.19–2.10 (m, 1H, H19), 1.89–1.61 (m, 3H, H18, H19); 1.53 (d, J_3,2_ = 7.1 Hz, 3H, H3); 1.22 (t, J_23,22_ = 7.1 Hz, 3H, H23); **^13^C NMR** (100 MHz, CDCl_3_) δ in ppm: 179.39 (C1); 172.87 (C21); 157.34 (C9); 137.68 (C4); 133.45 (C7); 129.26 (C11); 129.00 (C12); 126.82 (C6); 126.73 (C13); 125.81 (C5); 118.63 (C10); 105.54 (C8); 61.85 (C22); 58.40 (C20); 55.29 (C14); 46.78 (C2); 45.77 (C17); 29.77 (C19); 24.69 (C18); 18.89 (C3); 14.09 (C23); **FT-IR**: ν (ATR): 2971; 2938; 2909; 2647; 2562; 2514; 2399; 2284; 2229; 2192; 2162; 2152; 2140; 2128; 2112; 2037; 1741; 1630; 1603; 1565; 1504; 1480; 1453; 1414; 1369; 1346; 1332; 1304; 1268; 1253; 1228; 1206; 1169; 1120; 1090; 1060; 1020; 957; 925; 893; 879; 856; 815; 790; 779; 748; 692; 680; 661; 640; 475 cm^−1^. **UV-Vis** (EtOH): λ_max_ = 228.2 nm; **Elemental analysis:** Calc. (%) for CHNO (373.443 g/mol) C (67.541), H (7.287), N (3.751), O (21.421). Found C (66.169), H (7.354), N (4.124), O (20.729).

[ProOPr][NAP]–L-proline propyl ester naproxenate

L-proline propyl ester naproxenate was obtained as a white solid in a 97% yield.


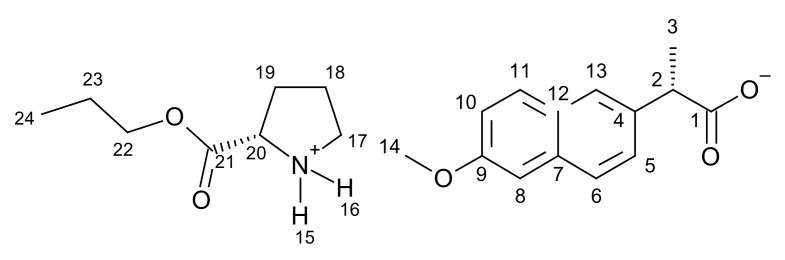


**^1^H NMR** (400 MHz, CDCl_3_) δ in ppm: 8.72 (s, 2H, H15, H16); 7.67–7.63 (s+d, 3H, H6, H8, H11); 7.46–7.41 (dd,1H, H10); 7.10–7.07 (s+d, 2H, H5, H13); 4.10–4.00 (m, 3H, H20, H22); 3.89 (s, 3H, H14); 3.79–3.74 (q, 1H, H2); 3.03 (t, J_18,17_ = 6.6 Hz, 2H, H17); 2.20–2.11 (m, 1H, H19), 1.90–1.65 (m, 3H, H18, H19); 1.64–1.59 (q, 2H, H23); 1.53 (d, J_3,2_ = 7.1 Hz, 3H, H3); 0.90 (t, J_24,23_ = 7.4 Hz, 3H, H24); **^13^C NMR** (100 MHz, CDCl_3_) δ in ppm: 179.32 (C1); 173.12 (C21); 157.35 (C9); 137.65 (C4); 133.46 (C7); 129.26 (C11); 129.01 (C12); 126.82 (C6); 126.73 (C13); 125.81 (C5); 118.62 (C10); 105.57 (C8); 67.33 (C22); 58.43 (C20); 55.28 (C14); 46.74 (C2); 45.78 (C17); 29.85 (C19); 24.73 (C18); 21.86 (C23); 18.88 (C3); 10.26 (C24); **FT-IR**: ν (ATR): 2968; 2940; 2895; 2881; 2555; 2362; 2323; 2241; 2231; 2222; 2209; 2193; 2166; 2151; 2137; 2098; 2026; 2019; 1740; 1630; 1603; 1578; 1504; 1480; 1454; 1437; 1382; 1364; 1348; 1304; 1270; 1254; 1209; 1190; 1180; 1168; 1117; 1095; 1061; 1038; 1020; 1006; 995; 940; 925; 894; 879; 857; 816; 791; 749; 692; 475 cm^−1^. **UV-Vis** (EtOH): λ_max_ = 228.1 nm; **Elemental analysis:** Calc. (%) for CHNO (387.469 g/mol) C (68.196), H (7.543), N (3.615), O (20.646). Found C (67.094), H (7.567), N (3.762), O (20.173).

[ProOiPr][NAP]–L-proline isopropyl ester naproxenate

L-proline isopropyl ester naproxenate was obtained as a white solid in a 99% yield.


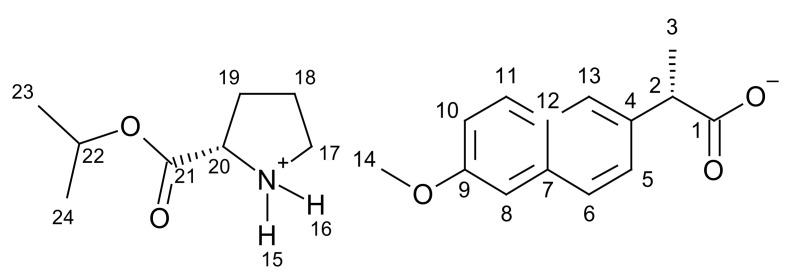


**^1^H NMR** (400 MHz, CDCl3) δ in ppm: 8.54 (s, 2H, H15, H16); 7.68–7.63 (s + d, 3H, H6, H8, H11); 7.46–7.44 (dd, 1H, H10); 7.11–7.07 (s + d, 2H, H5, H13); 5.04–4.97 (m, 1H, H22); 4.00–3.94 (m, 1H, H20); 3.89 (s, 3H, H14); 3.79–3.74 (q, 1H, H2); 3.03 (t, J_18,17_ = 6.8 Hz, 2H, H17); 2.20–2.10 (m, 1H, H19), 1.87–1.63 (m, 3H, H18, H19); 1.53 (d, J_3,2_ = 7.1 Hz, 3H, H3); 1.22–1.19 (dd, 6H, H23, H24); **^13^C NMR** (100 MHz, CDCl_3_) δ in ppm: 179.35 (C1); 172.65 (C21); 157.33 (C9); 137.75 (C4); 133.45 (C7); 129.27 (C11); 129.02 (C12); 126.82 (C6); 126.75 (C13); 125.80 (C5); 118.60 (C10); 105.57 (C8); 69.55 (C22); 58.55 (C20); 55.28 (C14); 46.81 (C2); 45.84 (C17); 29.89 (C19); 24.72 (C18); 21.66 (C23, C24); 18.91 (C3); **FT-IR**: ν (ATR): 2975; 2943; 2568; 2354; 2323; 2278; 2232; 2193; 2168; 2153; 2140; 2099; 1733; 1630; 1603; 1531; 1505; 1481; 1454; 1437; 1383; 1364; 1349; 1307; 1288; 1271; 1255; 1209; 1180; 1168; 1147; 1104; 1061; 1039; 1020; 996; 977; 958; 925; 908; 894; 880; 858; 844; 815; 792; 695; 671; 476 cm^−1^; **UV-Vis** (EtOH): λ_max_ = 228.2 nm; **Elemental analysis:** Calc. (%) for CHNO (387.469 g/mol) C (68.196), H (7.543), N (3.615), O (20.646). Found C (66.837), H (7.629), N (3.816), O (19.973).

[ProOBu][NAP]–L-proline butyl ester naproxenate

L-proline butyl ester naproxenate was obtained as a white solid in a 99% yield.


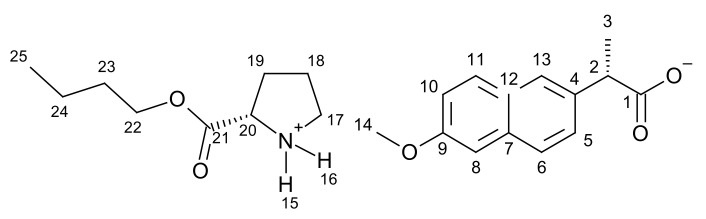


**^1^H NMR** (400 MHz, CDCl3) δ in ppm: 8.60 (s, 2H, H15, H16); 7.67–7.63 (s+d, 3H, H6, H8, H11); 7.45–7.43 (dd,1H, H10); 7.10–7.07 (s+d, 2H, H5, H13); 4.14–4.00 (m, 3H, H20, H22); 3.89 (s, 3H, H14); 3.79–3.74 (q, 1H, H2); 3.03 (t, J_18,17_ = 6.3 Hz, 2H, H17); 2.20–2.21 (m, 1H, H19), 1.89–1.63 (m, 3H, H18, H19); 1.61–1.56 (q, 2H, H23); 1.53 (d, J_3,2_ = 7.2 Hz, 3H, H3); 1.38–1.28 (m, 2H, H24); 0.91 (t, J_25,24_ = 7,4 Hz, 3H, H25); **^13^C NMR** (100 MHz, CDCl_3_) δ in ppm: 179.37 (C1); 173.03 (C21); 157.34 (C9); 137.69 (C4); 133.45 (C7); 129.26 (C11); 129.01 (C12); 126.82 (C6); 126.73 (C13); 125.81 (C5); 118.62 (C10); 105.54 (C8); 65.68 (C22); 58.41 (C20); 55.28 (C14); 46.78 (C2); 45.77 (C17); 30.48 (C23); 29.72 (C19); 24.70 (C18); 19.01 (C24); 18.90 (C3); 13.67 (C25); **FT-IR**: ν (ATR): 3064; 3030; 2955; 2934; 2898; 2873; 2659; 2363; 2343; 2226; 2210; 2180; 2169; 2152; 2098; 1733; 1630; 1602; 1506; 1485; 1473; 1456; 1440; 1417; 1388; 1354; 1341; 1306; 1286; 1265; 1232; 1177; 1159; 1126; 1075; 1057; 1031; 997; 960; 924; 905; 873; 849; 822; 794; 748; 696; 683; 653; 479 cm^−1^; **UV-Vis** (EtOH): λ_max_ = 228.0 nm; **Elemental analysis:** Calc. (%) for CHNO (401.496 g/mol) C (68.805), H (7.782), N (3.489), O (18.623). Found C (67.569), H (7.842), N (3.703), O (19.431).

#### 3.1.1. Comparison of NMR Spectra for [ProOR][NAP]

**Figure 1:**
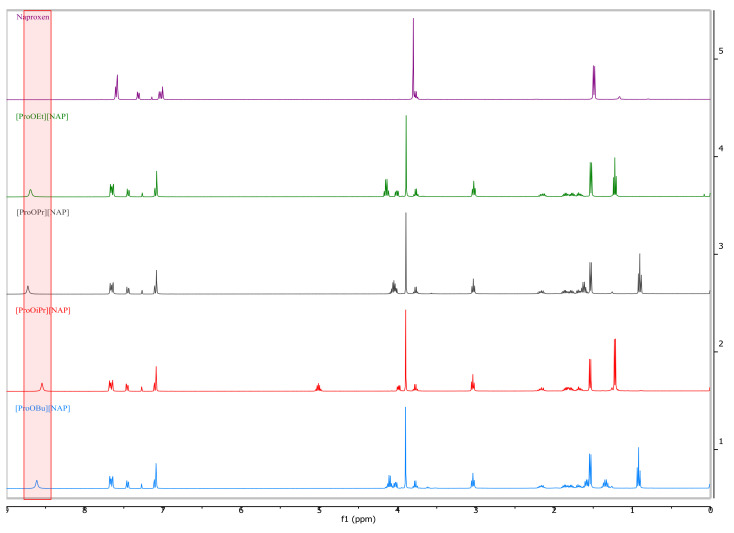
^1^H NMR spectra for naproxen (purple), [ProOEt][NAP] (green), [ProOPr][NAP] (black), [ProOiPr][NAP] (red), and [ProOBu][NAP] (blue) (the protonated amino group are marked in the red square).

NMR spectra were used primarily to identify and evaluate the purity of the obtained compounds. This method was also used to confirm the ionic structure. In the ^1^H NMR spectra, a characteristic broad singlet corresponds to two protons in the range between 8.54 ppm for [ProOiPr][NAP] to 8.72 ppm for [ProOPr][NAP]. A rapid intermolecular exchange partially causes the broadening. These signals correspond to protonated amine group in L-proline alkyl ester naproxenates (in Figure 1, these signals are marked in the red square). Detailed ^1^H NMR spectra with integrations and chemical shifts highlighted can be found in the Supplementary Data (Figures S1–S4).

**Figure 2:**
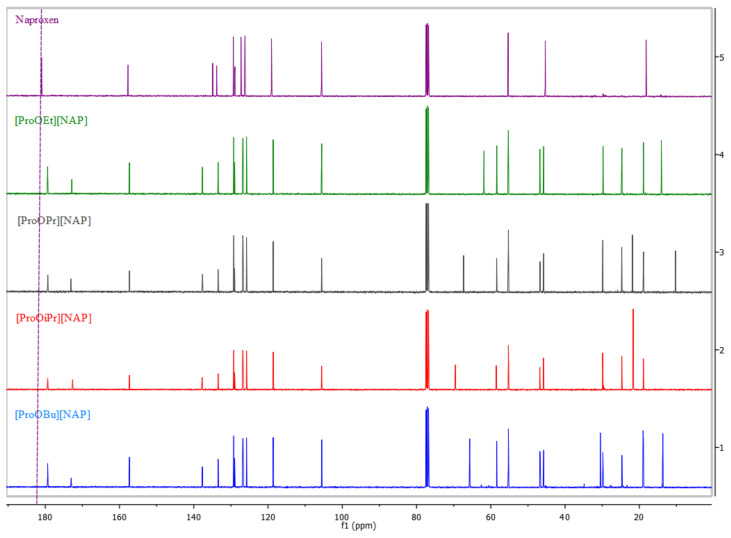
^13^C NMR spectra for naproxen (purple), [ProOEt][NAP] (green), [ProOPr][NAP] (black), [ProOiPr][NAP] (red), and [ProOBu][NAP] (blue) (carbonyl carbon shift in naproxen is highlighted in purple).

In the ^13^C NMR spectra, the lower chemical shift of the carbonyl carbon signal for L-proline alkyl ester naproxenates compared to that for the naproxen is another confirmation of the ionic structure of the obtained compounds. The difference between these chemical shifts is about 2 ppm. For the naproxen, this signal is localized at 181.00 ppm, while for [ProOEt][NAP], [ProOPr][NAP], [ProOiPr][NAP], and [ProOBu][NAP], they were 179.39, 179.37, 179.35, and 179.37 ppm, respectively [39,40,41,42]. In Figure 2, the shift of this signal in naproxen is marked in purple. Detailed ^13^C NMR spectra with the selected chemical shifts are in the Supplementary Data (Figure S5–S8).

#### 3.1.2. Comparison of FTIR Spectra for [ProOR][NAP]

**Figure 3:**
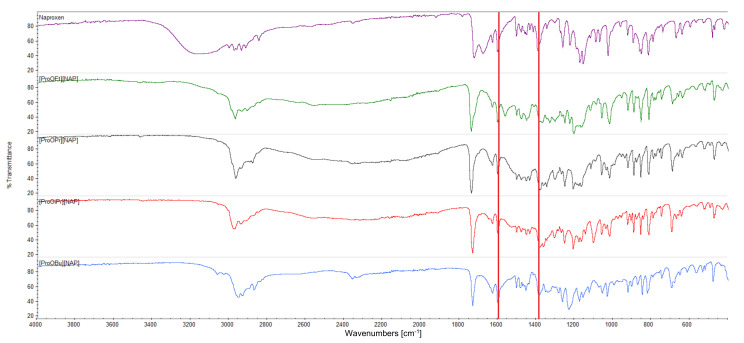
ATR-FTIR spectra for naproxen (purple), [ProOEt][NAP] (green), [ProOPr][NAP] (black), [ProOiPr][NAP] (red), [ProOBu][NAP] (blue) (ν_sym._) (COO^−^), and asymmetric (ν_asym._) (COO^−^) stretching vibrations marked in red).

The presence of characteristic absorption bands in the ATR-FTIR spectra also confirmed the identity of the obtained naproxen derivatives. In the ATR-FTIR spectra, the presence of strong bands at ca. 1570 and 1390 cm^−1^ additionally confirmed the ionic structure of the molecules obtained. These bands correspond, accordingly, to the symmetric (ν_sym._) (COO^−^) and asymmetric (ν_asym._) (COO^−^) stretching vibrations of the carboxylate anion. This shift and a difference above 200 cm^−1^ between the frequency values of these two bands confirmed the presence of the carboxylate anion COO^−^ and the ionic structure of L-proline alkyl ester naproxenates [43,44,45]. Figure 3 shows a comparison of the ATR-FTIR spectra of naproxen and the obtained L-proline alkyl ester naproxenates. Detailed ATR-FTIR spectra with the selected major absorption bands are in the Supplementary Data (Figure S9–S12). Figure 3 shows a comparison of the ATR-FTIR spectra of all compounds, where the bands confirming the ionic structure are marked in detail.

Moreover, analysis of the ATR-FTIR spectra confirmed that anhydrous salts were obtained.

The elemental analysis confirmed the content of individual elements (C, H, N, O) in synthesized L-proline alkyl ester naproxenates.

### 3.2. X-ray Powder Diffraction

Reflections that can be attributed to the naproxen phase ((2S)-2-(6-methoxynaphthalen-2-yl) propanoic acid (PDF 00-040-15550) were identified in the naproxen diffraction pattern.

**Figure 4:**
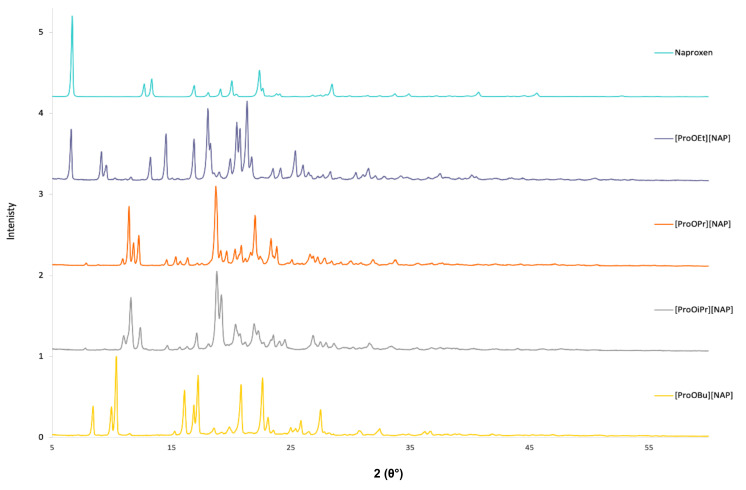
X-ray diffraction patterns of naproxen (lith blue), [ProOEt][NAP] (navy blue), [ProOPr][NAP] (orange), [ProOiPr][NAP] (gray), [ProOBu][NAP] (yellow).

The XRPD pattern shown in Figure 4 clearly indicated the crystalline nature for both unmodified naproxen and its salts with L-proline alkyl esters. Crystalline salts exhibit characteristic diffraction peaks, but each diffractogram is different. There are several sharp peaks in the diffraction pattern of salts, but they usually occur at different 2θ than for unmodified naproxen.

### 3.3. Physicochemical Properties of [ProOR][NAP]

**Figure 5:**
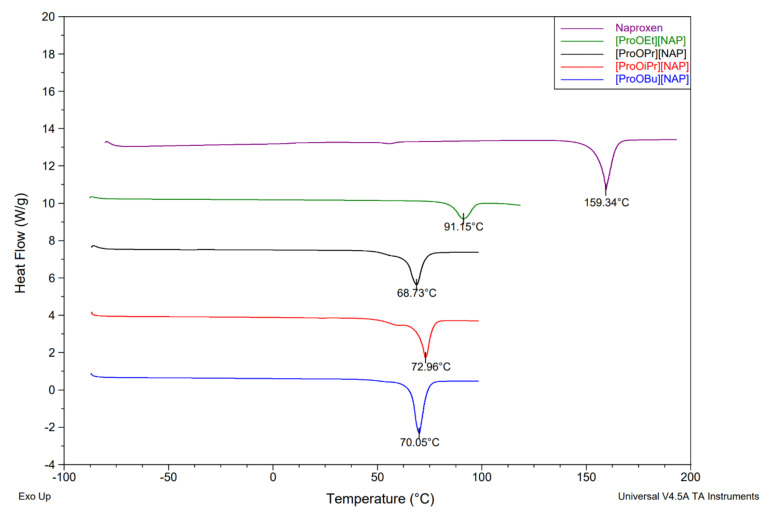
The DSC curves of naproxen (purple), L-proline alkyl ester naproxenates-[ProOEt][NAP] (green), [ProOPr][NAP] (black), [ProOiPr][NAP] (red), and [ProOBu][NAP] (blue).

The condition for classifying compounds to the group of ionic liquids is their ionic structure, which is confirmed in this case by spectroscopic methods, and the melting point below 100 °C [46,47,48,49,50,51,52,53]. The L-proline alkyl ester naproxenates are white solids at room temperature, and their melting points are summarized in Table 2. The melting point was determined from DSC curve analysis. Figure 5 shows a summary of the DSC curves from the first heating cycle for all analyzed compounds. In contrast, the DSC curves in the sample’s heating–cooling–heating–cooling–heating sequence are available in the Supplementary Data (Figure S13–S16). It was noted that the melting point depended on the alkyl chain length of the amino acid ester and decreased with increasing chain length. As in the case of the isopropyl ester, the branching of the alkyl chain caused an increase in the melting point compared to an n-propyl chain. These relationships are known and demonstrated in previous publications for ibuprofen, ketoprofen, and naproxen derivatives [33,34,39,48].

Our goal was to create salts with a melting point below 100 °C ionic liquids because the use of ionic liquids has advantages, such as avoiding the phenomenon of polymorphism. As can be seen, the formation of naproxen salts leads to lowering the melting point significantly (<80 °C), indicating lower crystal lattice energy and ultimately increasing the solubility. Lowering the melting point of compounds is associated with a particular risk during the technological processing during the preparation of the final drug form. However, due to the compounds’ properties, they can be successfully used as drugs applied to the skin, such as patches, ointments, or gels. Moreover, a melting point below 60 °C will not be a technological problem for these drug forms.

**Figure 6:**
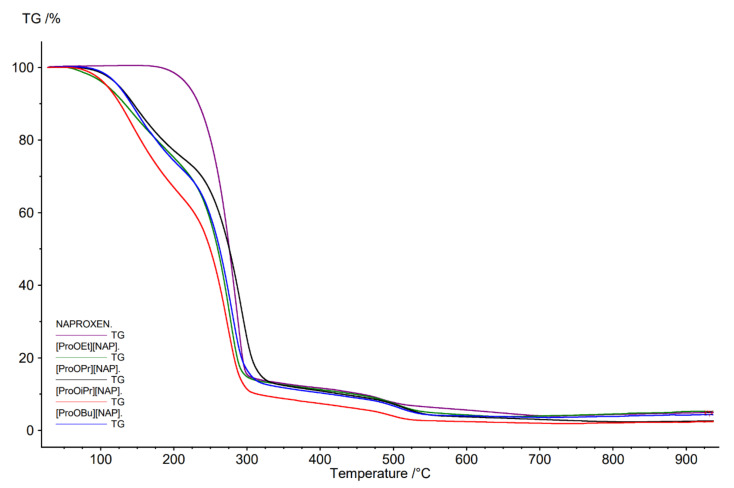
The TG curves of naproxen (purple), L-proline alkyl ester naproxenates-[ProOEt][NAP] (green), [ProOPr][NAP] (black), [ProOiPr][NAP] (red), and [ProOBu][NAP] (blue).

The thermal stability of naproxen and L-proline alkyl ester naproxenates was determined and compared (Figure 6). TG, DTG and c-DTA curves are in the Supplementary Data (Figure S17–S20). As with the L-leucine and L-valine derivatives [34], two steps of thermal weight loss were observed for the L-proline derivatives compared to one stage for naproxen. Table 2 summarizes the onset temperature of decomposition (T_onset_) and the maximum weight loss rate (T_max_) temperature for the investigated compounds. T_onset_ for the [AAOR][NAP] was within the range of 99.6 °C for [ProOiPr][NAP] to 114.0 °C for [ProOBu][NAP], whereas the value for naproxen was 249.9 °C. Naproxenates of L-proline alkyl esters showed lower thermal stability than the unmodified naproxen. As noted, thermal stability increased with the lengthening of the alkyl chain, while the branching of the chain caused a decrease in thermal stability. [ProOiPr][NAP] showed the lowest thermal stability, which was even lower than [ProOEt][NAP] (Figure 6).

**Figure 7:**
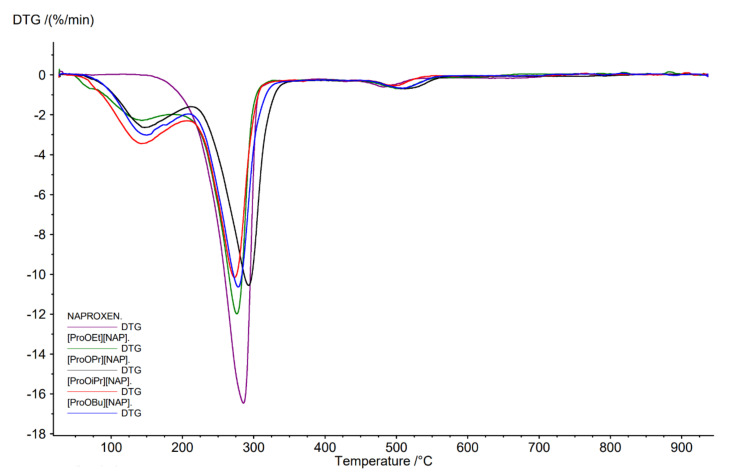
The DTG curves of naproxen (purple), and L-proline alkyl ester naproxenates-[ProOEt][NAP] (green), [ProOPr][NAP] (black), [ProOiPr][NAP] (red), and [ProOBu][NAP] (blue).

The maximum weight loss rate (T_max_) temperatures were similar for both naproxen and its derivatives (Table 2). [ProOEt][NAP] had the lowest T_max_, while naproxen was the compound with the highest T_max_ (Figure 7).

The lower thermal stability of the obtained naproxen salts can eliminate them from some dosage forms. Considering other advantages such as higher permeability and lower toxicity, we hope they are an alternative to the acid form of naproxen. In addition, the lower thermal stability causes the obtained compounds to be more easily degraded.

Additional stability tests should be performed for a new drug substance, particularly before registering such a drug. Evaluation of the effect of various environmental factors such as temperature, humidity, and light is particularly important for the storage conditions determination. For the new L-proline and naproxen derivatives, these studies will be continued in the future.

Since both the ionic liquid’s cation and anion have optical activity, the specific and molar rotation of the compounds obtained were determined. The results are summarized in Table 2. The specific rotation was similar for all obtained compounds, and it was found to be between +1.775 for [ProOiPr][NAP] and +5.660 for [ProOEt][NAP], as compared to +50.99 for naproxen and, and –82.84 for L-proline. It can be seen that the smaller share of naproxen mass in the total mass of its salt results in the lower values of the optical rotation. The exception is the derivative with the isopropyl chain, for which the rotation value was the lowest.

The pKa for naproxen was 4.15 [49], whereas the pKa values determined for L-proline alkyl esters were as follows: 9.63 for [ProOEt][NAP], 9.75 for [ProOiPr][NAP], 9.95 for [ProOPr][NAP], and 9.97 for [ProOBu][NAP]. The differences ΔpKa between naproxen pKa and L-proline alkyl esters pKa are greater than 5, which clearly indicates the formation of an ionized acid–base pair. It is known from the extensive studies on the dependence of the pharmaceuticals occurrence in ionized and non-ionized forms on the ΔpKa of the acid and base that ionized acid–base complexes are observed exclusively for ΔpKa > 4. However, for between −1 < ΔpKa < 4, non-ionized or ionized acid–base complexes can be expected, and the probability of the occurrence of ionized forms increases with increasing ΔpKa [50].

Our results clearly show that the salts of naproxen and L-proline alkyl esters are formed without difficulty.

Another value is pKs, which can be used to evaluate the strength of particular types of salt. Knowledge of the log(Ks) quantity also permits one to deduce the degree of disproportionation that would be anticipated if one were to dissolve salt in pure water.

For example, for [ProOEt][NAP], pKs = 4.15 + (14 − 9.63) − 14 = −5.48; and log(Ks) is 5.48, which indicates that the degree of salt formation would exceed 99%. All the obtained results are shown in Table 3 [51].

Due to the great importance of dissolution testing in drug discovery and development, we determined the solubility in water and typical organic solvents. The solubility data are summarized in Table 4. All L-proline alkyl ester naproxenates were soluble in ethanol, dimethyl sulfoxide, and chloroform, and they were all insoluble in water, ethyl acetate, diethyl ether toluene, and n-hexane. In addition, this study showed that in some apolar solvents, such as ethyl acetate and diethyl ether, the solubility was slightly worse, except for chloroform, in which the solubility of the tested naproxen derivatives was higher than naproxen.

Since aqueous solubility is one of the most influencing factors in the bioavailability of the drugs, we also assessed the solubility of the L-proline alkyl ester naproxenates in deionized water and phosphate-buffered saline solution. Aqueous solubility is also the main key to drug effectiveness. The results, expressed in g of compound per dm^3^ and the amount of active compound in g per dm^3^, are presented in Table 5. As can be seen, the solubility of L-proline alkyl ester naproxenates was markedly higher than naproxen, exceeding even 10 times higher (based on the concentration of active substance). The highest solubility in water was obtained for [ProOiP][NAP] (4.7646 ± 0.0050 [g dm^−3^]), while [ProOEt][NAP] had the lowest (2.3383 ± 0.0080 [g dm^−3^]). Interestingly, a rather unusual relationship between solubility and alkyl chain length was observed: solubility increased slightly with increasing chain length, and this phenomenon has not been explained. A similar dependence was observed for the solubility in PBS (pH 7.4) buffer solution. For all the tested compounds, the solubility in PBS (pH 7.4) buffer was markedly higher than in water, but these tests were conducted at a higher temperature (32 °C for PBS (pH 7.4), 25 °C for water).

The partition coefficients between n-octanol and water (log Po/w) were determined to predict drug hydrophobicity and partitioning in biological systems. The octanol–water partition coefficients for naproxen and L-proline alkyl ester naproxenates are presented in Table 6. All naproxenates showed positive log P values but were lower than that of naproxen, indicating that the formation of salts reduced hydrophobicity. However, longer alkyl chains in the ester group of L-proline yielded an increase in hydrophobicity: the log P in the octanol/water system for naproxenates increased from 0.898 to 1.094 when the alkyl chain was changed from ethyl to butyl.

**Figure 8:**
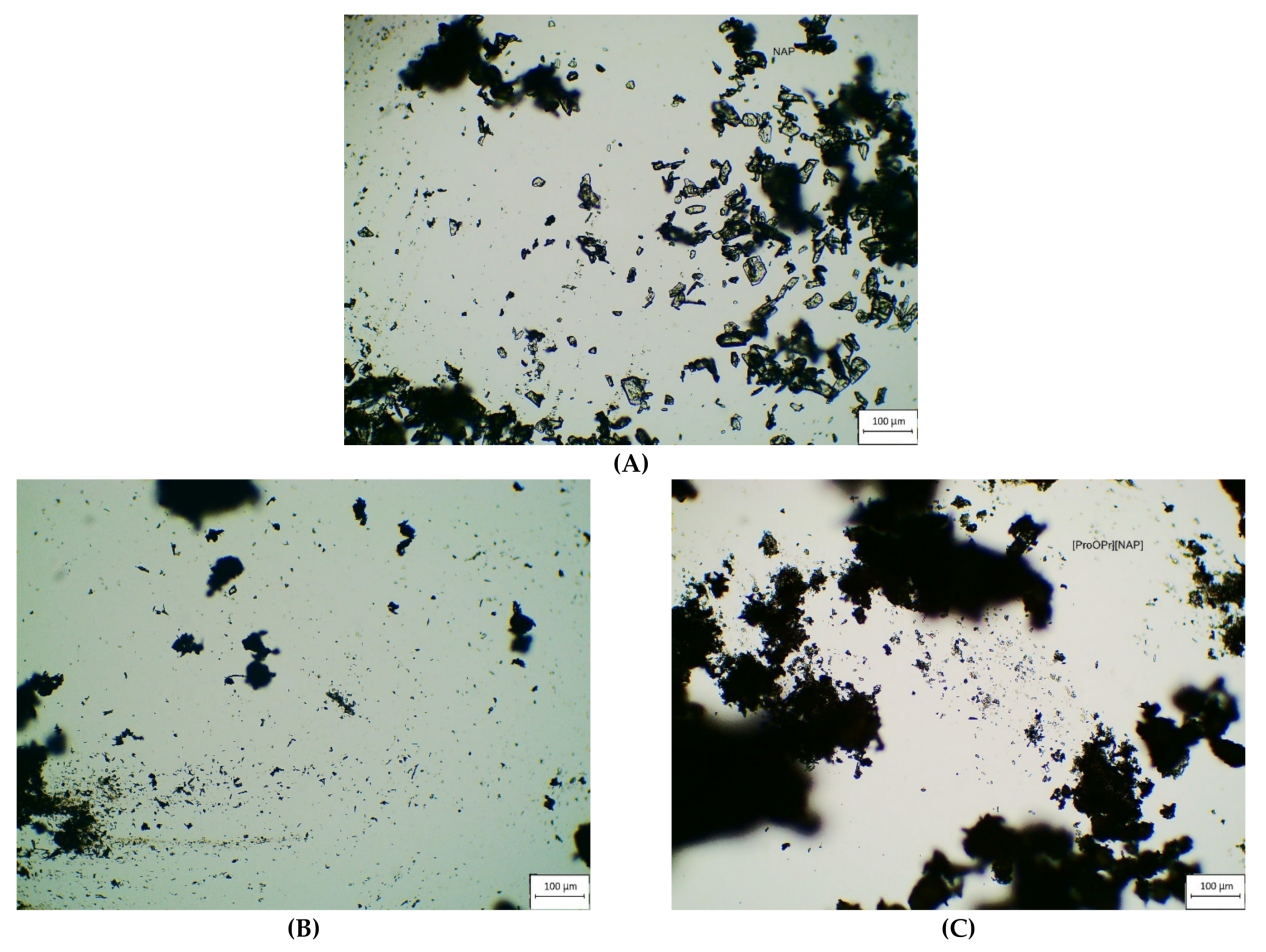

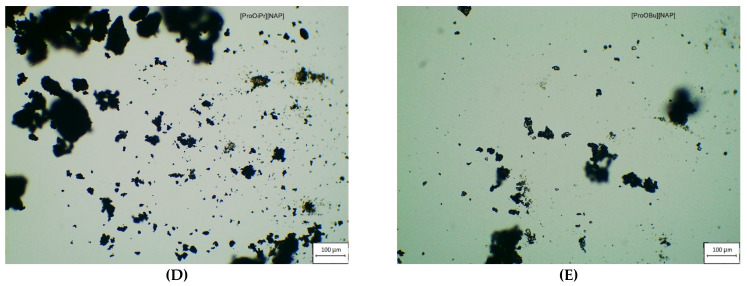
Micrographs of (**A**) [NAP], (**B**) [ProOEt][NAP], (**C**) [ProOPr][NAP], (**D**) [ProOiPr][NAP], and (**E**) [ProOBu][NAP].

The images of crystal habits of naproxen and its derivatives are presented in Figure 8. Spherical agglomerates are observed for both naproxen and its derivatives. Pure naproxen drug powder and [ProOPr][NAP] show rod-like crystals. Needle-like crystals were produced for [PreOEt][NAP], while a more cubic crystal habit was obtained for [PeOiPr][NAP] and [ProOBu][NAP].

### 3.4. Skin Permeation and Skin Accumulation of Naproxen and Its Salts with L-Proline Esters

Naproxen is a potent nonsteroidal anti-inflammatory drug (NSAID) used for various inflammatory conditions [52]. However, similar to other NSAIDs, oral naproxen’s most common side effect is gastrointestinal irritation [53]. One of the methods to reduce the side effect of naproxen is the topical/transdermal administration route [54]. Some anti-inflammatory drugs, including naproxenates, should permeate the skin quickly and show a rapid therapeutic effect [31,52]. However, the permeation of these substances is restricted by the stratum corneum, consisting mainly of lipids, namely cholesterol, ceramides, and fatty acids [55]. One method of increasing the permeation of drugs through the skin is modifying the active ingredient itself [39]. In the present study, the structural modification of naproxen consists of forming its ion pair, in which the counterion is the L-proline alkyl ester cation. The ion-pair method temporarily neutralizes the charge. The ion pairs easily penetrate the lipids of the stratum corneum and then dissociate in the lower layers of the epidermis [56,57,58]. A similar approach is known, among other things, in ibuprofen derivatives [59].

In our in vitro study using Franz diffusion cells, the permeation of new naproxen derivatives was compared with the permeation of free naproxen. The skin permeation studies were conducted using abdomen porcine skin. Porcine skin is frequently used for the preliminary evaluation of percutaneous permeation of topically applied drugs because histopathological studies have confirmed its similarity to human skin [26,27].

**Figure 9:**
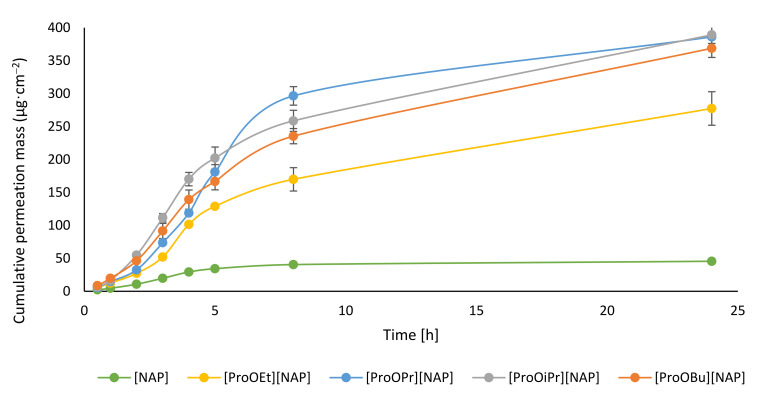
Time course of the cumulative mass of naproxen and its derivatives during the 24 h permeation. 1 cm^3^ of PBS (pH 7.4) containing a constant concentration of compounds was placed in each donor chamber (Mean ± SD, *n* = 3). Statistically significant difference was estimated using the ANOVA test.

**Figure 10:**
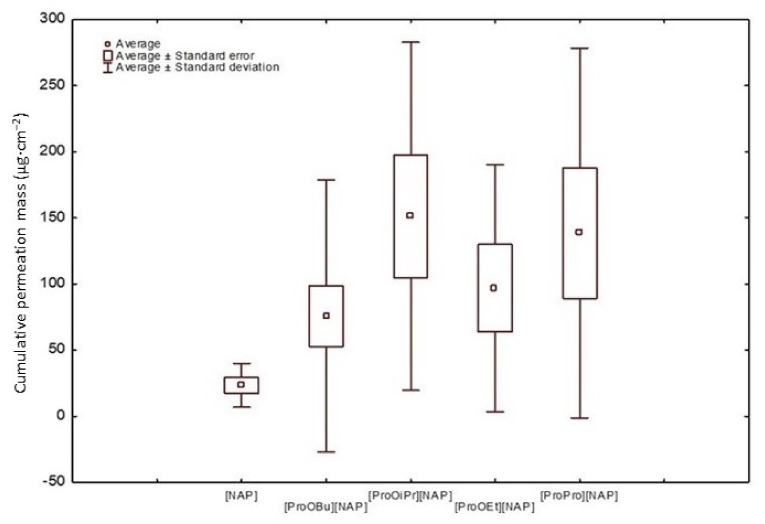
The box-plot of cumulative mass for naproxen and its derivatives after 24 h permeation.

**Figure 11:**
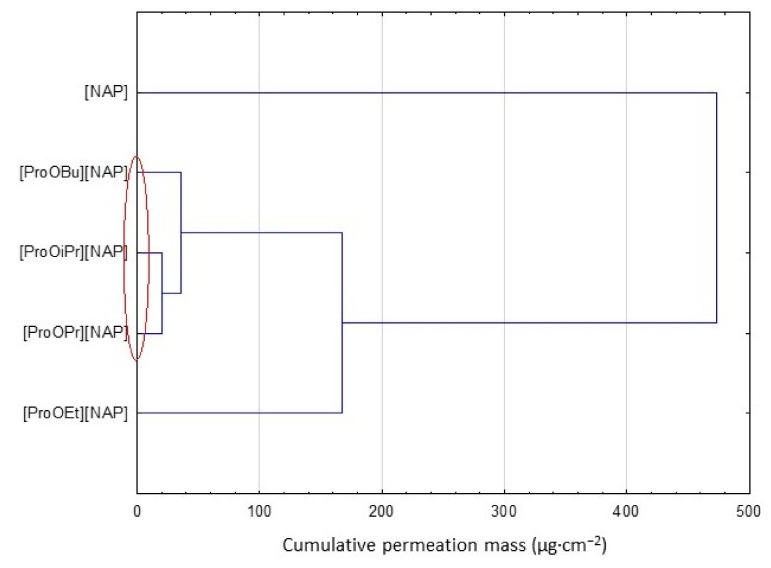
Cluster analysis graph for the mean accumulated mass of naproxen and its derivatives during the entire 24 h study. Compounds with similar values are marked with a red circle.

The cumulative mass of the tested compounds in acceptor fluid, considering all time points, is presented in Figure 9. Meanwhile, the content of naproxen and its derivatives in the acceptor fluid collected during 24 h permeation is summarized in Table 7. The cumulative mass of the individual compounds, determined after 24 h of permeation, was as follows: [ProOiPr][NAP] > [ProOPr][Nap] > [ProOBu][NAP] > [ProOEt][NAP] and NAP. The cumulative mass of all naproxen derivatives in the acceptor phase after 24 h of permeation was significantly higher than free naproxen. From among the studied derivatives, [ProOiPr][NAP] > [ProOPr][Nap] > [ProOBu][NAP] permeated to a higher degree than others; the cumulative amounts of substances permeated during the 24 h study were 389.12 ± 13.031, 386.02 ± 1.574, and 368.70 ± 13.848 µg, respectively (Table 7, Figure 10). These results are also confirmed by the cluster analysis test, in which these compounds form a separate cluster (Figure 11). The similarity between derivatives was also found using the Mann–Whitney test, which showed a statistically significant difference between all derivatives and pure naproxen (*p* < 0.001) (Table 8).

The results of the in vitro efficiency permeation experiments related to naproxen and L-proline alkyl ester naproxenates are summarized in Table 9. The permeation parameters such as flux, apparent permeability coefficient, lag time, diffusion coefficient in the skin, skin partition coefficient, percent drug permeated after 24 h, and enhancement factor were calculated. A marked difference in the flux for the salified form of the naproxen and the form of its salts was immediately evident: the transdermal flux of the L-proline alkyl ester naproxenate PBS (pH 7.4) solution was from 2.80 to 3.83 times higher than that of the 2.5% naproxen suspension in PBS (pH 7.4) (21.854 for [ProOEt][NAP], 29.753 for [ProOiPr][NAP] vs. 7.778 µg cm^−2^ h^−1^, respectively). The permeability coefficient (K_P_), a quantitative measure of the rate at which a molecule can cross the skin, was also determined. K_P_ is composed of factors related to both the drug and the barrier and their interaction. This parameter eliminates the effect of compound concentration. For the tested compounds, K_P_ values were from 2.682 to 3.936 times higher than for naproxen. The lag time was significantly increased by introducing a structural modification of naproxen and increasing its hydrophobicity: L-proline alkyl ester naproxenate resulted in a higher flux value after a longer lag time than unmodified naproxen. The diffusion coefficient in the skin was approximately 2.911 and 5.294 cm^2^ h^−1^ for [ProOPr][NAP] and [ProOBu][NAP], respectively (Table 9). This value was considerably lower than 9.066 cm^2^ h^−1^ for 2.5% naproxen suspension in PBS (pH 7.4). Another important parameter is K_m_, which describes the drug’s ability to escape from the solution and move into the outermost layers of the stratum corneum; it is defined as the equilibrium solubility of the drug in the stratum corneum relative to its solubility in the vehicle. In this study, Km values ranged from 0.950 to 2.104 for [ProOBu][NAP] and [ProOPr][NAP] respectively, compared to the much higher value of 9.066 for naproxen. These results suggest that the compounds under study can promote repartition in the skin.

**Figure 12:**
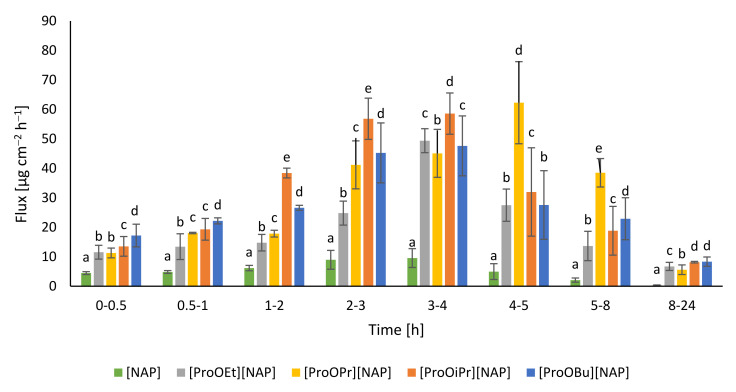
The permeation rate of naproxen and its derivatives during the 24 h permeation; different letters indicate significant differences between the tested compounds; (the letter ‘a’ stands for the compounds with the lowest permeation in a given time point, while the letter ‘e’—the compounds with the highest permeation at a given time point); α = 0.05 (Mean ± SD, *n* = 3). Statistically significant difference was estimated using the ANOVA test.

For drugs NSAIDs, faster permeation through the skin is preferable to achieve a rapid therapeutic effect. Increased permeation in less time causes a faster decrease in inflammation in the underlying tissues [48]. The permeation rate determined at each time interval is presented in Figure 12. The highest permeation rate to the acceptor fluid was observed in samples collected between 2 and 4 h for [ProOiPr][NAP] and [ProOBu][NAP] and between 4 and 5 h for [ProOEt][NAP]. We have shown in previous studies that ibuprofen pairing with L-valine alkyl esters caused greater drug molecule permeation through pig skin [36]. Likewise, Sarveiya et al., showed a higher steady-state flow of ibuprofen triethylammonium salt through a PDMS membrane with pH 7.0 buffer as the acceptor phase compared to sodium ibuprofenate [60].

**Figure 13:**
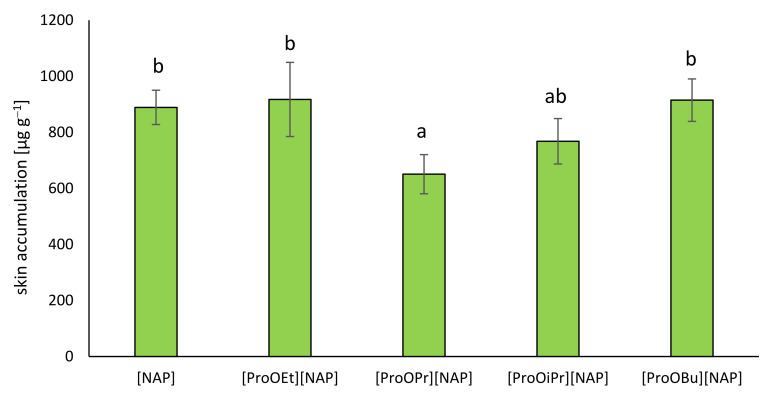
Accumulation in the skin of naproxen and its derivatives during the 24 h penetration; different letters indicate significant differences between the tested compounds; (the letter “a” stands for compounds accumulating in the smallest amount in the skin, while the letter “b”—compounds accumulating in the greatest amount in the skin); α = 0.05 (mean ± SD, *n* = 3). Statistically significant difference was estimated using the ANOVA test.

Importantly, drugs may both permeate and accumulate in the skin. For topical delivery systems, accumulation in the skin with low permeation is desired, while for transdermal delivery systems, the opposite behavior is preferred [61]. In the case of anti-inflammatory drugs, faster and greater permeation is most often preferred, with less accumulation in the skin. Figure 13 shows the mass of naproxen and its derivatives accumulated in porcine skin after 24 h of penetration. All the compounds used accumulated in the skin. The lowest accumulation values were obtained for [ProOPr][NAP] and [ProOiPr][NAP]: 650.62 ± 69.83 and 768.06 ± 81.05, respectively (Figure 13).

### 3.5. Cytotoxicity of Naproxen and Its Salts

**Figure 14:**
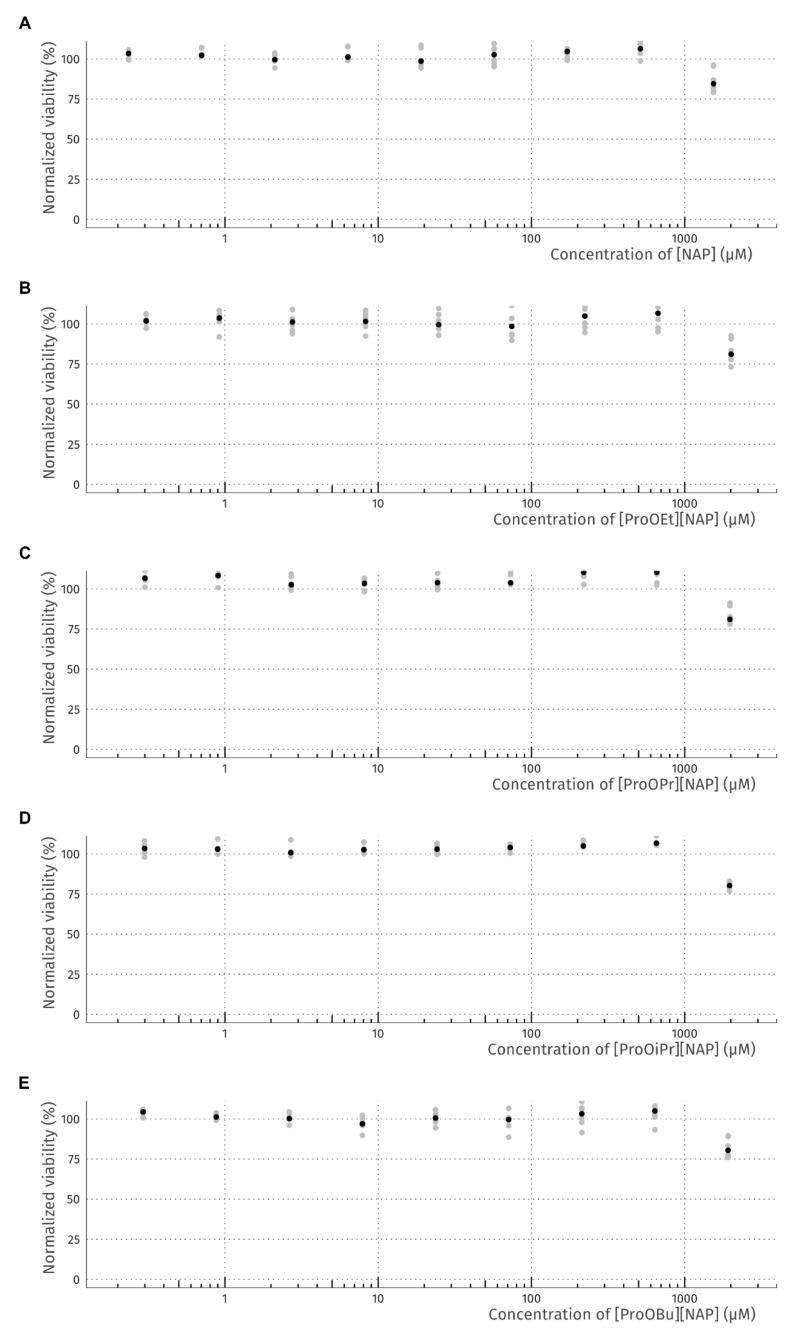
L929 cell viability (24 h exposure) as a function of dose for: (**A**) [NAP], (**B**) [ProOEt][NAP], (**C**) [ProOPr][NAP], (**D**) [ProOiPr][NAP], and (**E**) [ProOBu][NAP]. Gray dots represent technical replicates; black dots represent medians.

In order to assess the potential cytotoxicity of the obtained compounds, a dose–response study was performed using a murine fibroblast cell line (L929). For all tested compounds, a modest reduction in viability, to 80–83%, was observed at the highest dose (≈2 mM) (Figure 14B–E). This effect was similar to that of NAP, where a reduction in viability to 87% was observed for the highest dose (≈1.5 mM) (Figure 14A). For the case of all dilutions, no adverse effect on viability was observed. Importantly, even in the case of the highest dose, where a reduction in viability was observed relative to vehicle alone, robust growth was still observed (see representative micrographs, Figure S21). Likewise, the morphology of the cells was normal, and no debris was observed. Collectively, these results point to a modest growth inhibitory effect rather than cytotoxicity. This effect can likely be attributed to the fact that naproxen is a COX inhibitor and thus influences arachidonic acid metabolism, which plays a key role in cell proliferation and survival [62]. Additionally, naproxen also influences polyamine biosynthesis, and doses similar to those used here (1–2 mM) were shown to cause growth inhibition, rather than loss of viability, in human colorectal cancer cells after 48 h incubation [63].

## 4. Conclusions

Four new salts, L-proline alkyl ester naproxenates of the C2–C4 chain, previously undescribed in the literature, were obtained via solvent-free synthesis. The obtained compounds showed improved solubility in water and PBS (pH 7.4) buffer solution simulating body fluids, as well as lower lipophilicity than naproxen. Notably, the obtained naproxen derivatives show increased permeability of the active substance through porcine skin, from 2.7 to over 3.9 times greater. The highest permeability was observed for naproxen with L-proline propyl and isopropyl ester, which also had higher transport rates and the greatest mass of permeating naproxen. Changes in these properties may increase the bioavailability of naproxen by various routes of administration. In addition, we showed that the length of the alkyl ester chain affects the permeability of the active substance. Finally, in vitro cell culture study indicated a similar effect—modest growth inhibition at the highest concentration—on murine fibroblast viability as the starting NSAID, naproxen. The presented results suggest that the obtained derivatives could offer a superior alternative to naproxen, especially in preparations applied to the skin.

## Figures and Tables

**Table 1:** Yields of the synthesized L-proline alkyl ester naproxenates [ProOR][NAP].

Compound	Yield (%)	State
[ProOEt][NAP]	98.33 ± 0.47	white solid
[ProOPr][NAP]	98.00 ± 0.82	white solid
[ProOiPr][NAP]	97.67 ± 0.94	white solid
[ProOBu][NAP]	98.67 ± 0.47	white solid

**Table 2:** The melting points, thermal stability, specific and molar rotation of naproxen and L-proline alkyl ester naproxenates.

Compound	Molar Mass (g mol^−1^)	T_m_ (°C)	T_onset_ (°C)	T_max_ (°C)	Specific Rotation ${\left[\alpha \right]}_{D}^{20}$	Molar Specific Rotation ${\left[M\right]}_{D}^{20}$
Naproxen	230.260	156.37	249.9	280.7	+50.99	+117.41
[ProOEt][NAP]	373.443	91.15	107.3	273.7	+5.660	+21.137
[ProOPr][NAP]	387.469	70.05	108.4	290.1	+4.200	+16.274
[ProOiPr][NAP]	387.469	72.96	99.6	273.0	+1.775	+6.878
[ProOBu][NAP]	401.496	68.73	114.0	276.4	+2.740	+11.001

T_m_—melting point; T_onset_—the onset of the thermal degradation; [*α*]*_λ_^T^*—specific rotation; [*M*]*_λ_^T^*—molar specific rotation.

**Table 3:** Predicted degree of salts formation for L-proline alkyl ester naproxenates [ProOR][NAP].

Compound	pKa of the Acid	Log(Ks)	Percent Salt Formed
[ProOEt][NAP]	9.63	5.48	>99
[ProOPr][NAP]	9.95	5.80	>99
[ProOiPr][NAP]	9.75	5.60	>99
[ProOBu][NAP]	9.97	5.82	>99

**Table 4:** Solubility in water and organic solvents of naproxen and L-proline alkyl ester naproxenates at 25 °C.

Compound	Water (63.1)	Ethanol (51.9)	DMSO (45.1)	Chloroform (39.1)	Ethyl Acetate (38.1)	Diethyl Ether (34.5)	Toluene (33.9)	n-Hexane (31.0)
Naproxen	-	+	+	+/-	+/-	+/-	-	-
[ProOEt][NAP]	-	+	+	+	-	-	-	-
[ProOPr][NAP]	-	+	+	+	-	-	-	-
[ProOiPr][NAP]	-	+	+	+	-	-	-	-
[ProOBu][NAP]	-	+	+	+	-	-	-	-

+ soluble >100 g dm^−3^; +/− partly soluble 33–100 g dm^−3^; —practically insoluble < 33 g dm^−3^ at 25 °C; in brackets solvent polarity parameter E_T_(30) are given.

**Table 5:** The solubility of naproxen and L-proline alkyl ester naproxenates in water at 25 °C and PBS (pH 7.4) buffer at 32 °C.

Compound	Solubility in Water at 25 °C		Solubility in PBS (pH 7.4) at 32 °C	
	g dm^−^^3^	g NAP dm^−3^	g dm^−3^	g NAP dm^−3^
Naproxen	0.2699 ± 0.0008	0.2699 ± 0.0008	1.6936 ± 0.0036	1.6936 ± 0.0036
[ProOEt][NAP]	3.7924 ± 0.0129	2.3383 ± 0.0080	5.113 ± 0.0209	3.153 ± 0.1289
[ProOPr][NAP]	4.1099 ± 0.0103	2.4424 ± 0.0061	5.742 ± 0.0080	3.412 ±0.0048
[ProOiPr][NAP]	4.7646 ± 0.0050	2.8314 ± 0.0030	5.249 ± 0.0319	3.119 ± 0.0190
[ProOBu][NAP]	4.4698 ± 0.0050	2.5635 ± 0.0029	5.995 ± 0.0765	3.438 ± 0.0439

**Table 6:** The n-octanol-water partition coefficient (log P) of naproxen and L-proline alkyl ester naproxenates.

Compound	logP
Naproxen	2.117 ± 0.0097
[ProOEt][NAP]	0.898 ± 0.0148
[ProOPr][NAP]	0.987 ± 0.0189
[ProOiPr][NAP]	0.921 ± 0.0373
[ProOBu][NAP]	1.094 ± 0.0218

**Table 7:** Cumulated mass for naproxen and its derivatives; different letters indicate significant differences between the tested compounds, α = 0.05, mean ± SD, *n* = 3. Statistically significant difference was estimated using the ANOVA test.

**Cumulative Permeation Mass (µg·cm^−2^)**	**[NAP]**	**[ProOEt][NAP]**	**[ProOPr][NAP]**	**[ProOiPr][NAP]**	**[ProOBu][NAP]**
	45.55 ± 1.89 ^a^	277.38 ± 25.438 ^b^	386.02 ± 1.574 ^c^	389.12 ± 13.031 ^c^	368.70 ± 13.848 ^c^

**Table 8:** Significant differences in the cumulative mass between all analyzed compounds, taking into account all time points during the entire 24 h permeation, were estimated by the Mann–Whitney test.

	[NAP]	[ProOEt][NAP]	[ProOPr][Nap]	[ProOiPr][NAP]	[ProOBu][NAP]
**[NAP]**		Z = −3.3081 *p* = 0.000 *	Z = −3.3081 *p* = 0.000 *	Z = −3.3081 *p* = 0.000 *	Z = −3.3081 *p* = 0.000 *
**[ProOEt][NAP]**			Z = −0.4725 *p* = 0.6363	Z = 1.5228 *p* = 0.1278	Z = −0.4725 *p* = 0.6363
**[ProOPr][Nap]**				Z = 0.2625 *p* = 0.7928	Z = −0.0525 *p* = 0.9581
**[ProOiPr][NAP]**					Z = 0.2625 *p* = 0.7928

* Value is significantly different (*p* < 0.05).

**Table 9:** Skin permeation parameters for naproxen and L-proline alkyl ester naproxenates; *n* = 3.

Compound	J_SS_, µg cm^−2^ h^−1^	K_P_∙10^3^, cm/h	L_T_, h	D∙10^4^, cm^2^/h	K_m_	Q%_24 h_	EF
Naproxen	7.778	3.111	0.460	9.066	0.172	1.822	1
[ProOEt][NAP]	21.854	8.345	1.316	3.167	1.318	6.531	2.682
[ProOPr][NAP]	29.158	12.247	1.431	2.911	2.104	9.635	3.936
[ProOiPr][NAP]	29.753	11.457	0.809	5.149	1.113	8.905	3.683
[ProOBu][NAP]	23.448	10.056	0.787	5.294	0.950	9.069	3.232

J_SS_—steady-state flux; K_P_—permeability coefficient; L_T_—lag time; D—diffusion coefficient; K_m_—skin partition coefficient; Q—the percentage of the applied dose, EF— enhancement factor.

## Data Availability

The data presented in this study are available on request from the corresponding author.

## Supplementary Materials

The following are available online at https://www.mdpi.com/article/10.3390/pharmaceutics13122110/s1, Figure S1: ^1^H NMR spectra of L-proline ethyl ester naproxenate, Figure S2: ^13^C NMR spectra of L-proline ethyl ester naproxenate, Figure S3: ^1^H NMR spectra of L-proline propyl ester naproxenate, Figure S4: ^13^C NMR spectra of L-proline propyl ester naproxenate, Figure S5: ^1^H NMR spectra of L-proline isopropyl ester naproxenate, Figure S6: ^13^C NMR spectra of L-proline isopropyl ester naproxenate, Figure S7: 1H NMR spectra of L-proline butyl ester naproxenate, Figure S8: ^13^C NMR spectra of L-proline butyl ester naproxenate, Figure S9: ATR-FTIR spectra of L-proline ethyl ester naproxenate, Figure S10: ATR-FTIR spectra of L-proline propyl ester naproxenate, Figure S11: ATR-FTIR spectra of L-proline isopropyl ester naproxenate, Figure S12: ATR-FTIR spectra of L-proline butyl ester naproxenate, Figure S13: The TG, DTG and c-DTA curves of L-proline ethyl ester naproxenate, Figure S14: The TG, DTG and c-DTA curves of L-proline propyl ester naproxenate, Figure S15: The TG, DTG and c-DTA curves of L-proline isopropyl ester naproxenate, Figure S16: The TG, DTG and c-DTA curves of L-proline butyl ester naproxenate, Figure S17: The DSC curves of L-proline ethyl ester naproxenate, Figure S18: The DSC curves of L-proline propyl ester naproxenate, Figure S19: The DSC curves of L-proline isopropyl ester naproxenate, Figure S20: The DSC curves of L-proline butyl ester naproxenate, Figure S21. Representative micrographs of L929 cells. (A) 24 h after seeding. (B) After further incubation for 24 h with vehicle. Panels C–L present row-wise pairs of L929 cells incubated for 24 h with the highest dose (right) and three-fold dilution (left) of tested compounds. C, D: [NAP]; E, F: [ProOEt][NAP]; G, H: [ProOPr][NAP]; I, J: [ProOiPr][NAP]; K, L: [ProOBu][NAP] Scale bar represents 200 µm. Method validation (linearity, sensitivity, accuracy and precision). Reference [64] is cited in the Supplementary Materials.

## References

[1] Haznar-Garbacz D., Polak S. (2011). Physiological factors affecting the bioavailability of the drugs. Farm. Pol..

[2] Currie G.M. (2018). Pharmacology, Part 2: Introduction to Pharmacokinetics. J. Nucl. Med. Technol..

[3] Nikinmaa M., Nikinmaa M. (2014). Factors Affecting the Bioavailability of Chemicals. An Introduction to Aquatic Toxicology.

[4] Atkinson A.J., Huang S.-M., Lertora J., Atkinson A. (2007). Drug Absorption and Bioavailability. Principles of Clinical Pharmacology.

[5] Rannou F., Pelletier J.-P., Martel-Pelletier J. (2016). Efficacy and Safety of Topical NSAIDs in the Management of Osteoarthritis: Evidence from Real-Life Setting Trials and Surveys. Semin. Arthritis Rheum..

[6] Newa M., Bhandari K.H., Kim J.O., Im J.S., Kim J.A., Yoo B.K., Woo J.S., Choi H.G., Yong C.S. (2008). Enhancement of Solubility, Dissolution and Bioavailability of Ibuprofen in Solid Dispersion Systems. Chem. Pharm. Bull..

[7] Hartlieb K.J., Ferris D.P., Holcroft J.M., Kandela I., Stern C.L., Nassar M.S., Botros Y.Y., Stoddart J.F. (2017). Encapsulation of Ibuprofen in CD-MOF and Related Bioavailability Studies. Mol. Pharm..

[8] Chow D.D., Karara A.H. (1986). Characterization, Dissolution and Bioavailability in Rats of Ibuprofen-β-Cyclodextrin Complex System. Int. J. Pharm..

[9] Li D.X., Oh Y.-K., Lim S.-J., Kim J.O., Yang H.J., Sung J.H., Yong C.S., Choi H.-G. (2008). Novel Gelatin Microcapsule with Bioavailability Enhancement of Ibuprofen Using Spray-Drying Technique. Int. J. Pharm..

[10] Yong C.S., Yang C.H., Rhee J.-D., Lee B.-J., Kim D.-C., Kim D.-D., Kim C.-K., Choi J.-S., Choi H.-G. (2004). Enhanced Rectal Bioavailability of Ibuprofen in Rats by Poloxamer 188 and Menthol. Int. J. Pharm..

[11] Ghosh L.K., Ghosh N.C., Chatterjee M., Gupta B.K. (1998). Product Development Studies on the Tablet Formulation of Ibuprofen to Improve Bioavailability. Drug Dev. Ind. Pharm..

[12] Irvine J., Afrose A., Islam N. (2018). Formulation and Delivery Strategies of Ibuprofen: Challenges and Opportunities. Drug Dev. Ind. Pharm..

[13] Zhang P., Forsgren J., Strømme M. (2014). Stabilisation of Amorphous Ibuprofen in Upsalite, a Mesoporous Magnesium Carbonate, as an Approach to Increasing the Aqueous Solubility of Poorly Soluble Drugs. Int. J. Pharm..

[14] Kumar L., Suhas B., Pai G.K., Verma R. (2015). Determination of Saturated Solubility of Naproxen Using UV Visible Spectrophotometer. Res. J. Pharm. Technol..

[15] Aboul-Fadl T., Al-Hamad S.S., Lee K., Li N., Gary B.D., Keeton A.B., Piazza G.A., Abdel-Hamid M.K. (2014). Novel Non-Cyclooxygenase Inhibitory Derivatives of Naproxen for Colorectal Cancer Chemoprevention. Med. Chem. Res..

[16] Jensen K., Löbmann K., Rades T., Grohganz H. (2014). Improving Co-Amorphous Drug Formulations by the Addition of the Highly Water Soluble Amino Acid, Proline. Pharmaceutics.

[17] Kasten G., Lobo L., Dengale S., Grohganz H., Rades T., Löbmann K. (2018). In Vitro and in Vivo Comparison between Crystalline and Co-Amorphous Salts of Naproxen-Arginine. Eur. J. Pharm. Biopharm..

[18] Mura P. (2003). Ternary Systems of Naproxen with Hydroxypropyl-β-Cyclodextrin and Aminoacids. Int. J. Pharm..

[19] Mura P., Bettinetti G.P., Cirri M., Maestrelli F., Sorrenti M., Catenacci L. (2005). Solid-State Characterization and Dissolution Properties of Naproxen–Arginine–Hydroxypropyl-β-Cyclodextrin Ternary System. Eur. J. Pharm. Biopharm..

[20] Marrucho I.M., Branco L.C., Rebelo L.P.N. (2014). Ionic Liquids in Pharmaceutical Applications. Annu. Rev. Chem. Biomol. Eng..

[21] Seddon K.R. (1997). Ionic Liquids for Clean Technology. J. Chem. Technol. Biotechnol..

[22] Gardas R.L., Coutinho J.A.P. (2009). Group Contribution Methods for the Prediction of Thermophysical and Transport Properties of Ionic Liquids. AIChE J..

[23] Azevedo A.M.O., Costa S.P.F., Dias A.F.V., Marques A.H.O., Pinto P.C.A.G., Bica K., Ressmann A.K., Passos M.L.C., Araújo A.R.T.S., Reis S. (2017). Anti-Inflammatory Choline Based Ionic Liquids: Insights into Their Lipophilicity, Solubility and Toxicity Parameters. J. Mol. Liq..

[24] Li P., Yin Y.-L., Li D., Woo Kim S., Wu G. (2007). Amino Acids and Immune Function. Br. J. Nutr..

[25] Wu G., Bazer F.W., Burghardt R.C., Johnson G.A., Kim S.W., Knabe D.A., Li P., Li X., McKnight J.R., Satterfield M.C. (2011). Proline and Hydroxyproline Metabolism: Implications for Animal and Human Nutrition. Amino Acids.

[26] Khiao In M., Richardson K.C., Loewa A., Hedtrich S., Kaessmeyer S., Plendl J. (2019). Histological and Functional Comparisons of Four Anatomical Regions of Porcine Skin with Human Abdominal Skin. Anat. Histol. Embryol..

[27] Jacobi U., Kaiser M., Toll R., Mangelsdorf S., Audring H., Otberg N., Sterry W., Lademann J. (2007). Porcine Ear Skin: An in Vitro Model for Human Skin. Ski. Res. Technol..

[28] Badran M.M., Kuntsche J., Fahr A. (2009). Skin Penetration Enhancement by a Microneedle Device (Dermaroller^®^) in Vitro: Dependency on Needle Size and Applied Formulation. Eur. J. Pharm. Sci..

[29] Haq A., Goodyear B., Ameen D., Joshi V., Michniak-Kohn B. (2018). Strat-M^®^ Synthetic Membrane: Permeability Comparison to Human Cadaver Skin. Int. J. Pharm..

[30] Simon A., Amaro M.I., Healy A.M., Cabral L.M., de Sousa V.P. (2016). Comparative Evaluation of Rivastigmine Permeation from a Transdermal System in the Franz Cell Using Synthetic Membranes and Pig Ear Skin with in Vivo-in Vitro Correlation. Int. J. Pharm..

[31] Ossowicz P., Klebeko J., Janus E., Nowak A., Duchnik W., Kucharski Ł., Klimowicz A. (2020). The Effect of Alcohols as Vehicles on the Percutaneous Absorption and Skin Retention of Ibuprofen Modified with l -Valine Alkyl Esters. RSC Adv..

[32] Davies D.J., Ward R.J., Heylings J.R. (2004). Multi-Species Assessment of Electrical Resistance as a Skin Integrity Marker for in Vitro Percutaneous Absorption Studies. Toxicol. Vitr..

[33] Ossowicz P., Kardaleva P., Guncheva M., Klebeko J., Świątek E., Janus E., Yancheva D., Angelov I. (2020). Ketoprofen-Based Ionic Liquids: Synthesis and Interactions with Bovine Serum Albumin. Molecules.

[34] Ossowicz P., Janus E., Klebeko J., Światek E., Kardaleva P., Taneva S., Krachmarova E., Rangelov M., Todorova N., Guncheva M. (2020). Modulation of the Binding Affinity of Naproxen to Bovine Serum Albumin by Conversion of the Drug into Amino Acid Ester Salts. J. Mol. Liq..

[35] Furniss B.S., Vogel A.I. (2009). Vogel’s Textbook of Practical Organic Chemistry.

[36] International Organization for Standardization (2009). ISO 10993-5:2009 Biological Evaluation of Medical Devices—Part 5: Tests for in Vitro Cytotoxicity.

[37] Holbeck S.L., Collins J.M., Doroshow J.H. (2010). Analysis of Food and Drug Administration–Approved Anticancer Agents in the NCI60 Panel of Human Tumor Cell Lines. Mol. Cancer Ther..

[38] Riss T.L., Moravec R.A., Niles A.L., Duellman S., Benink H.A., Worzella T.J., Minor L., Markossian S., Grossman A., Brimacombe K., Arkin M., Auld D., Austin C.P., Baell J., Chung T.D.Y., Coussens N.P., Dahlin J.L. (2004). Cell Viability Assays. Assay Guidance Manual.

[39] Janus E., Ossowicz P., Klebeko J., Nowak A., Duchnik W., Kucharski Ł., Klimowicz A. (2020). Enhancement of Ibuprofen Solubility and Skin Permeation by Conjugation with L-Valine Alkyl Esters. RSC Adv..

[40] Ossowicz P., Janus E., Schroeder G., Rozwadowski Z. (2013). Spectroscopic Studies of Amino Acid Ionic Liquid-Supported Schiff Bases. Molecules.

[41] Rozwadowski Z. (2005). Deuterium Isotope Effects on 13C Chemical Shifts of Lithium Salts of Schiff Bases Amino Acids. J. Mol. Struct..

[42] Breitmaier E., Voelter W. (1978). 13C-NMR Spectroscopy, Methods and Applications in Organic Chemistry. Monographs in Modern Chemistry.

[43] Vairam S., Premkumar T., Govindarajan S. (2010). Trimellitate Complexes of Divalent Transition Metals with Hydrazinium Cation: Thermal and Spectroscopic Studies. J. Therm. Anal. Calorim..

[44] Kolev T., Spiteller M., Koleva B. (2010). Spectroscopic and Structural Elucidation of Amino Acid Derivatives and Small Peptides: Experimental and Theoretical Tools. Amino Acids.

[45] Katritzky A.R. (1996). A Guide to the Complete Interpretation of Infrared Spectra of Organic Structures. N.P.G. Roeges. J. Am. Chem. Soc..

[46] Shamshina J.L., Zavgorodnya O., Rogers R.D., Davies P., Manisha K. (2019). Ionic Liquids. Encyclopedia of Analytical Science. Chemistry Molecular Sciences and Engineering.

[47] Lei Z., Chen B., Koo Y.-M., MacFarlane D.R. (2017). Introduction: Ionic Liquids. Chem. Rev..

[48] Singh S.K., Savoy A.W. (2020). Ionic Liquids Synthesis and Applications: An Overview. J. Mol. Liq..

[49] Jedynak K., Szczepanik B., Rędzia N., Słomkiewicz P., Kolbus A., Rogala P. (2019). Ordered Mesoporous Carbons for Adsorption of Paracetamol and Non-Steroidal Anti-Inflammatory Drugs: Ibuprofen and Naproxen from Aqueous Solutions. Water.

[50] Cruz-Cabeza A.J. (2012). Acid-base crystalline complexes and the pKa rule. Cryst. Eng. Comm..

[51] Brittain H.G. (2007). Strategy for the Prediction and Selection of Drug Substance Salt Forms. Pharm. Technol..

[52] Welton T. (2018). Ionic Liquids: A Brief History. Biophys. Rev..

[53] Disasa Irge D. (2016). Ionic Liquids: A Review on Greener Chemistry Applications, Quality Ionic Liquid Synthesis and Economical Viability in a Chemical Processes. AJPC.

[54] Akbari J., Saeedi M., Morteza-Semnani K., Rostamkalaei S.S., Asadi M., Asare-Addo K., Nokhodchi A. (2016). The Design of Naproxen Solid Lipid Nanoparticles to Target Skin Layers. Colloids Surf. B Biointerfaces.

[55] Escobar-Chávez J.J., Quintanar-Guerrero D., Ganem-Quintanar A. (2005). In Vivo Skin Permeation of Sodium Naproxen Formulated in Pluronic F-127 Gels: Effect of Azone^®^ and Transcutol^®^. Drug Dev. Ind. Pharm..

[56] Zillich O.V., Schweiggert-Weisz U., Hasenkopf K., Eisner P., Kerscher M. (2013). Release and in Vitro Skin Permeation of Polyphenols from Cosmetic Emulsions. Int. J. Cosmet. Sci..

[57] Atta-ur-Rahman W., Caldwell G., Iqbal Choudhary M., Yan Z. (2012). Frontiers in Drug Design &amp Discovery (Volume 4).

[58] Benson H. (2005). Transdermal Drug Delivery: Penetration Enhancement Techniques. CDD.

[59] Morrow D.I.J., McCarron P.A., Woolfson A.D., Donnelly R.F. (2007). Innovative Strategies for Enhancing Topical and Transdermal Drug Delivery. TODDJ.

[60] Sarveiya V., Templeton J.F., Benson H.A.E. (2010). Ion-Pairs of Ibuprofen: Increased Membrane Diffusion. J. Pharm. Pharmacol..

[61] Touitou E., Meidan V.M., Horwitz E. (1998). Methods for Quantitative Determination of Drug Localized in the Skin. J. Control. Release.

[62] Wang B., Wu L., Chen J., Dong L., Chen C., Wen Z., Hu J., Fleming I., Wang D.W. (2021). Metabolism Pathways of Arachidonic Acids: Mechanisms and Potential Therapeutic Targets. Sig. Transduct. Target. Ther..

[63] Hughes A., Saunders F.R., Wallace H.M. (2012). Naproxen Causes Cytotoxicity and Induces Changes in Polyamine Metabolism Independent of Cyclo-Oxygenase Expression. Toxicol. Res..

[64] ICH Q2 (R1) Validation of Analytical Procedures: Text and Methodology. https://www.ema.europa.eu/en/ich-q2-r1-validation-analytical-procedures-text-methodology.

