# Supplementary material for: Novel Naproxen Salts with Increased Skin Permeability
Source: Pharmaceutics. 2021 Dec 7;13(12):2110. doi: 10.3390/pharmaceutics13122110 (PMC8704648; doi:10.3390/pharmaceutics13122110)
Supplement: Supplementary file 1 [file pharmaceutics-13-02110-s001.zip › pharmaceutics-1450550-supplementary.pdf]

pharmaceutics

MDPI

# Supplementary Materials: Novel Naproxen Salts with Increased Skin Permeability

**Ewelina Świątek, Paula Ossowicz-Rupniewska, Ewa Janus, Anna Nowak, Peter Sobolewski, Wiktoria Duchnik, Łukasz Kucharski and Adam Klimowicz**

Number of pages: 14
Number of Figures: 21

## Table of Contents


1. The NMR spectra of [ProOR][NAP] 1-5
2. The ATR-FTIR spectra of [ProOR][NAP] 5-7
3. The TG curves of [ProOR][NAP] 7-9
4. The DSC curves of [ProOR][NAP] 9-11
5. Representative micrographs of L929 cells 12
6. Method Validation 13-14


## The NMR spectra of [ProOR][NAP]

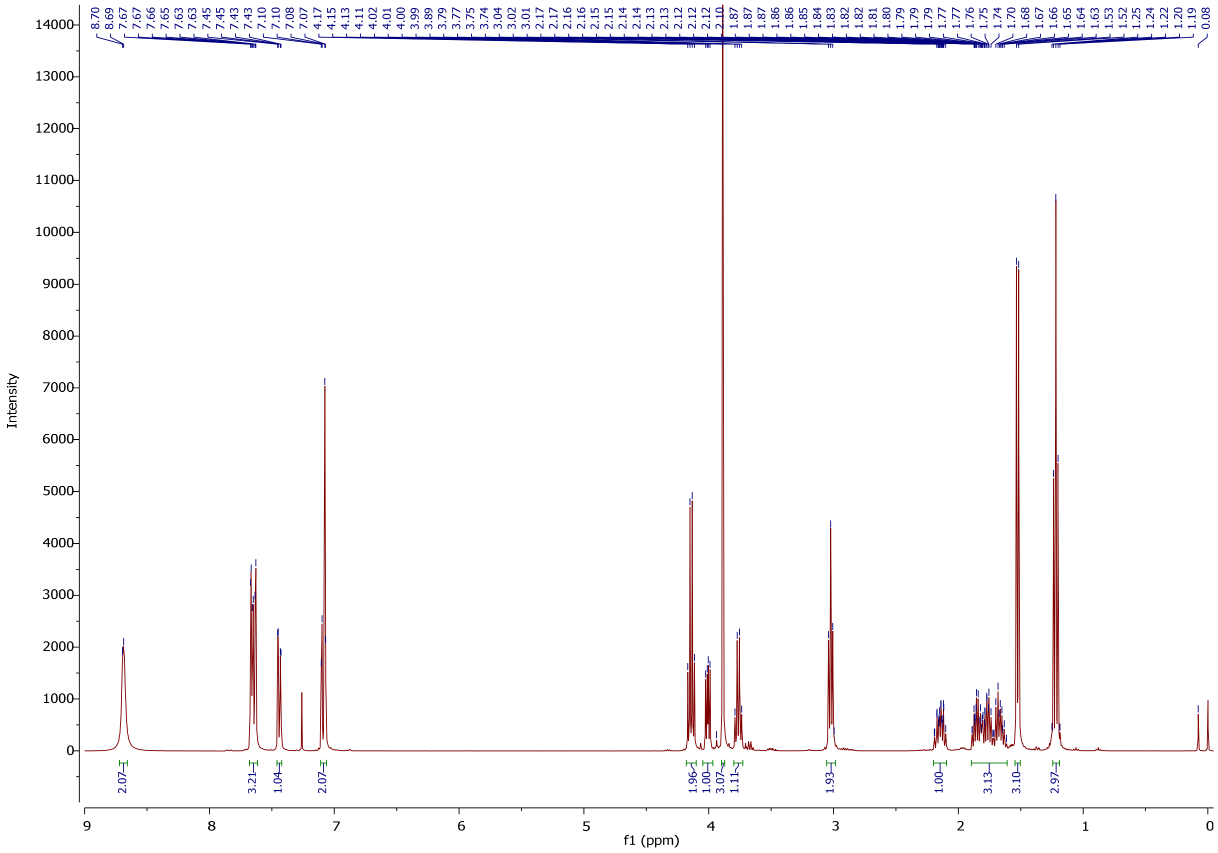


**Figure S1.** $^1$H NMR spectra of L-proline ethyl ester naproxenate.

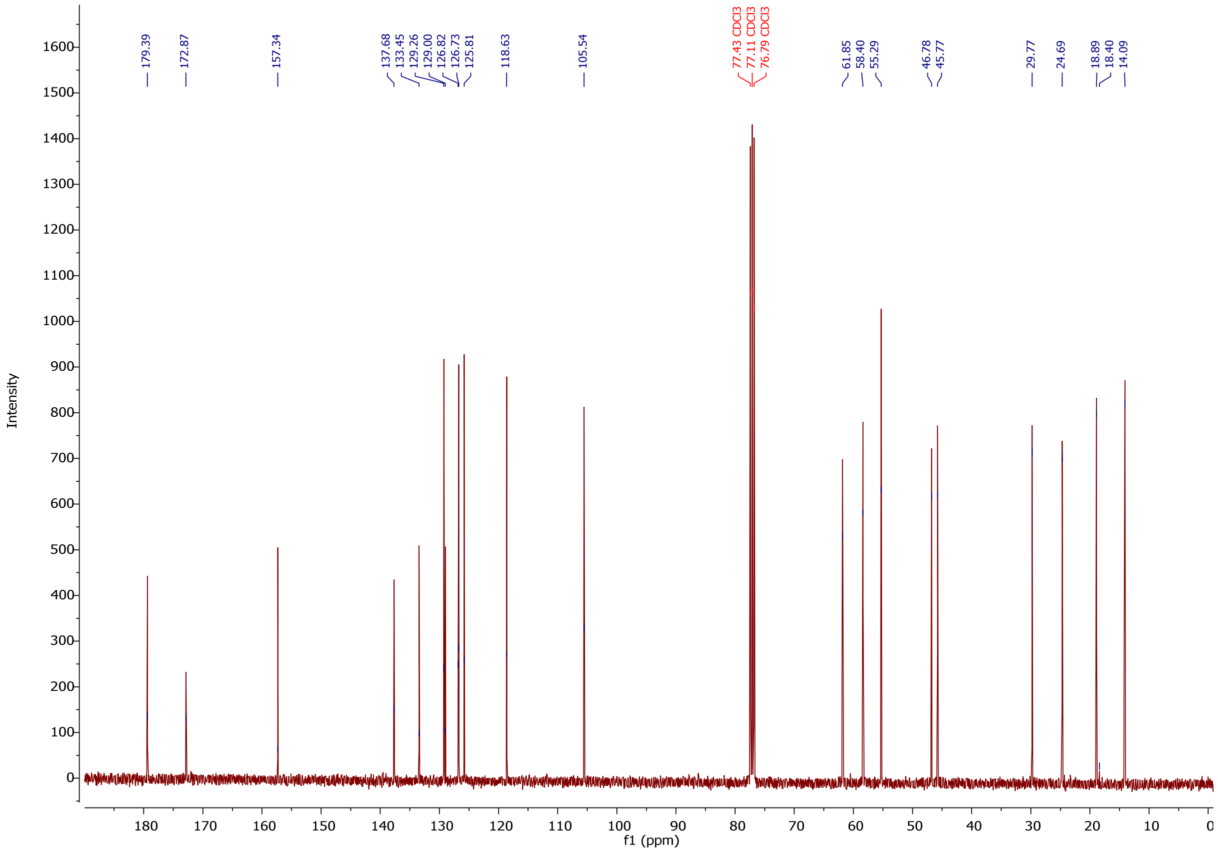

**Figure S2.** $^{13}$C NMR spectra of L-proline ethyl ester naproxenate.

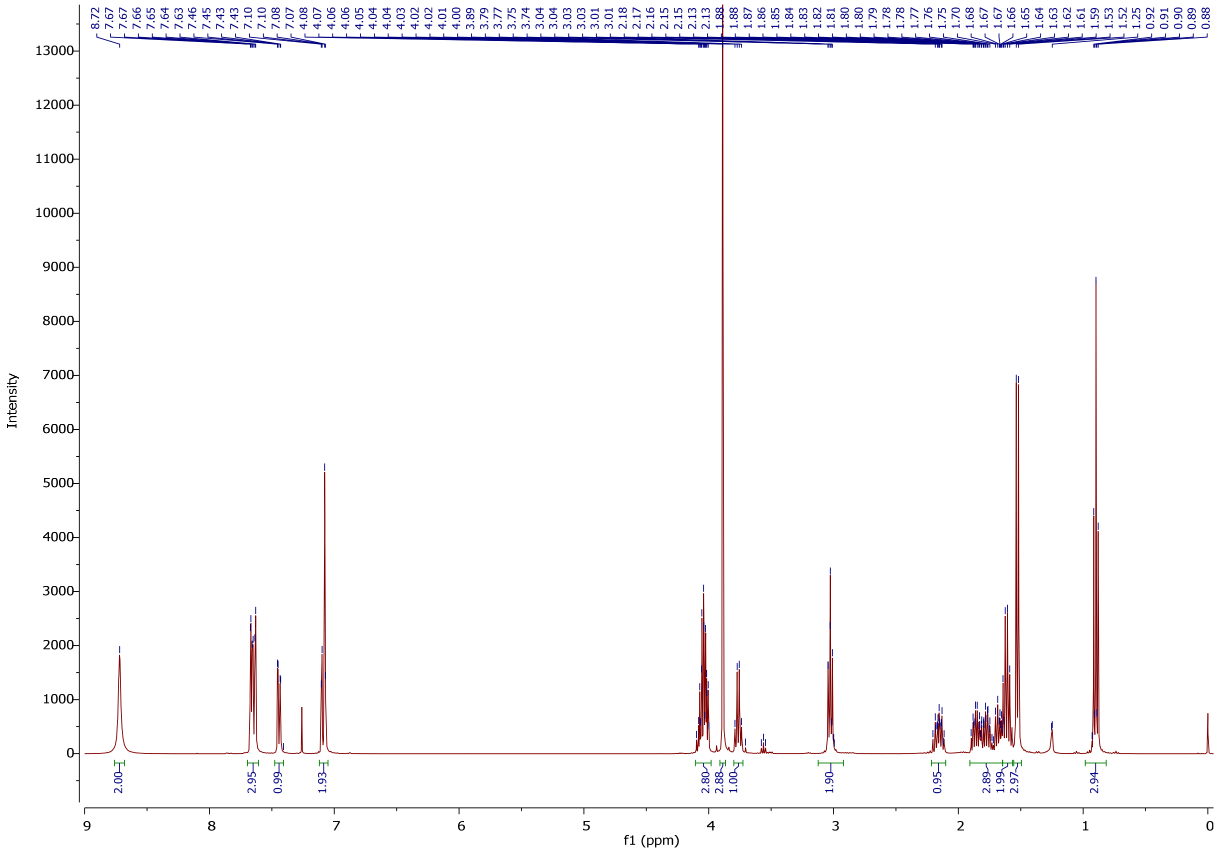

**Figure S3.** $^{1}$H NMR spectra of L-proline propyl ester naproxenate.

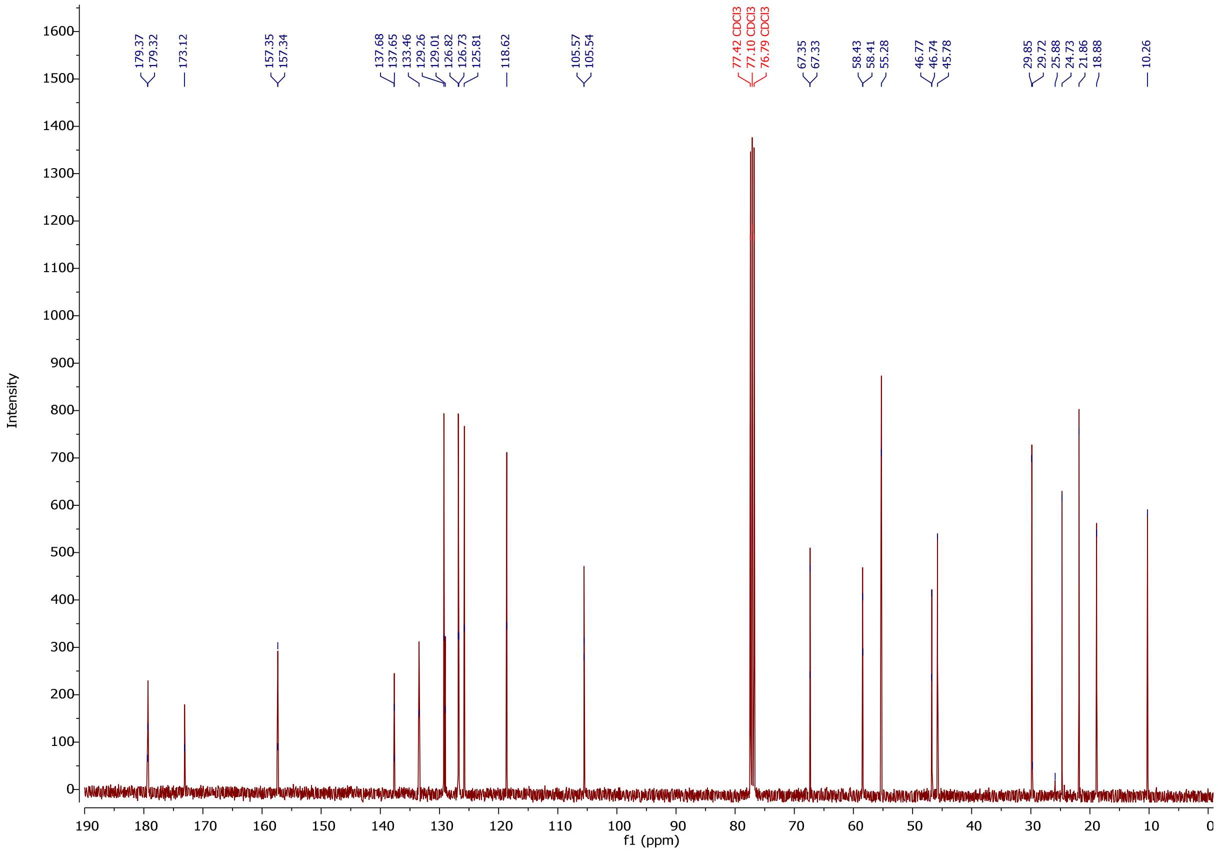


**Figure S4.** $^{13}C$ NMR spectra of L-proline propyl ester naproxenate.

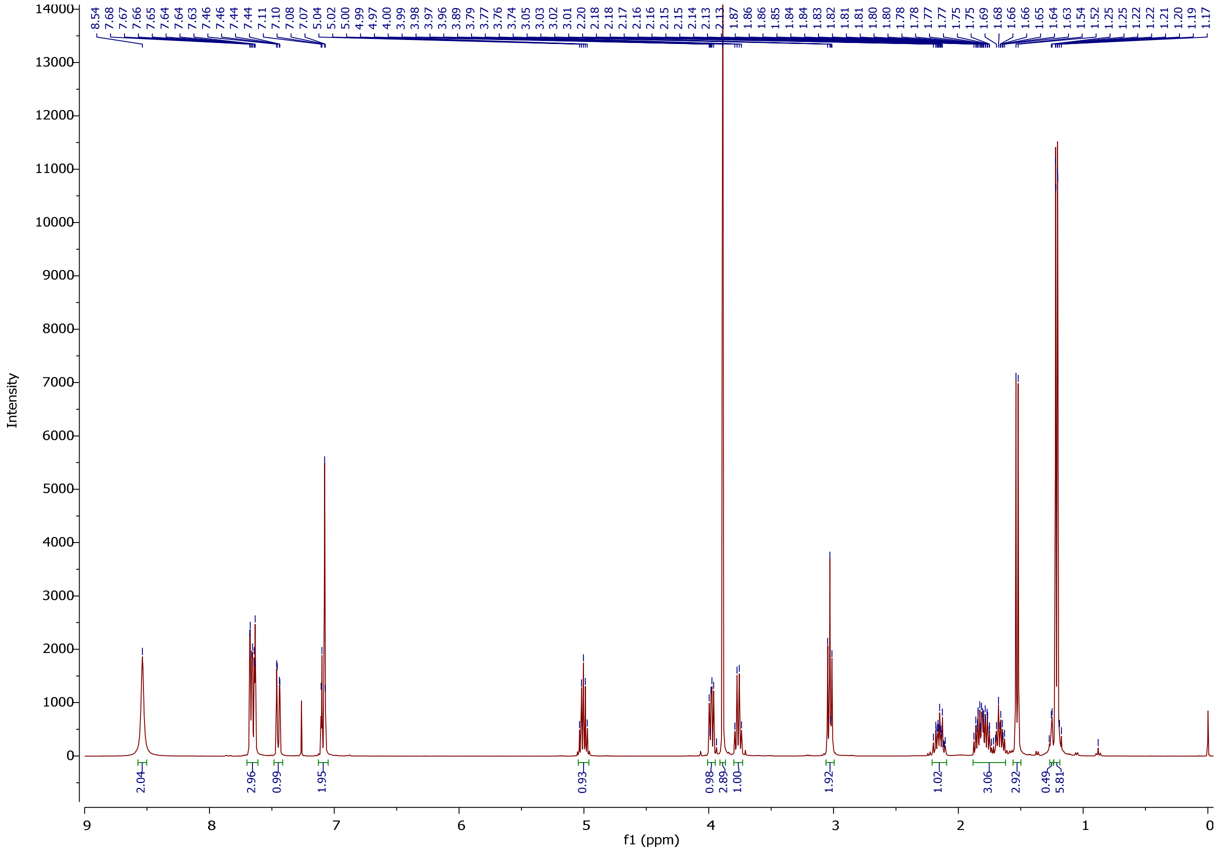


**Figure S5.** $^{1}H$ NMR spectra of L-proline isopropyl ester naproxenate.

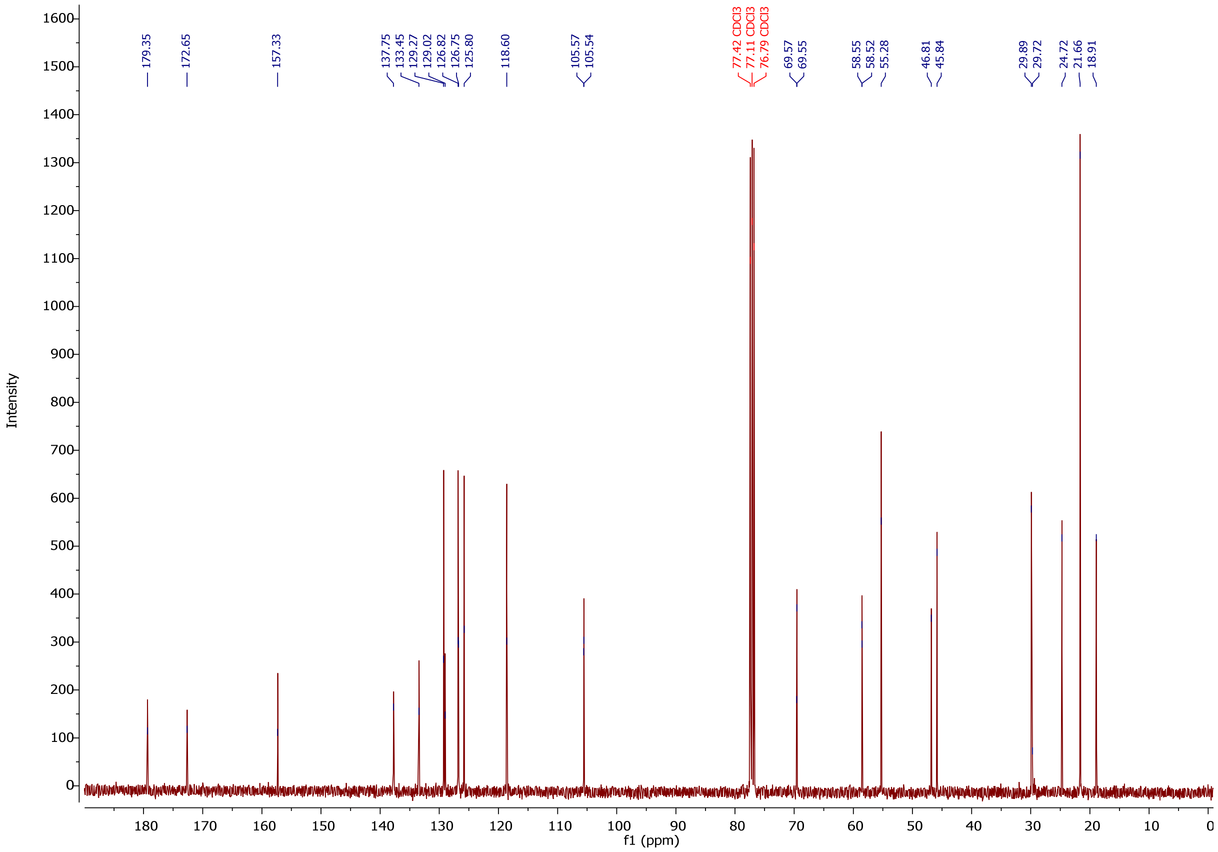

**Figure S6.** $^{13}C$ NMR spectra of L-proline isopropyl ester naproxenate.

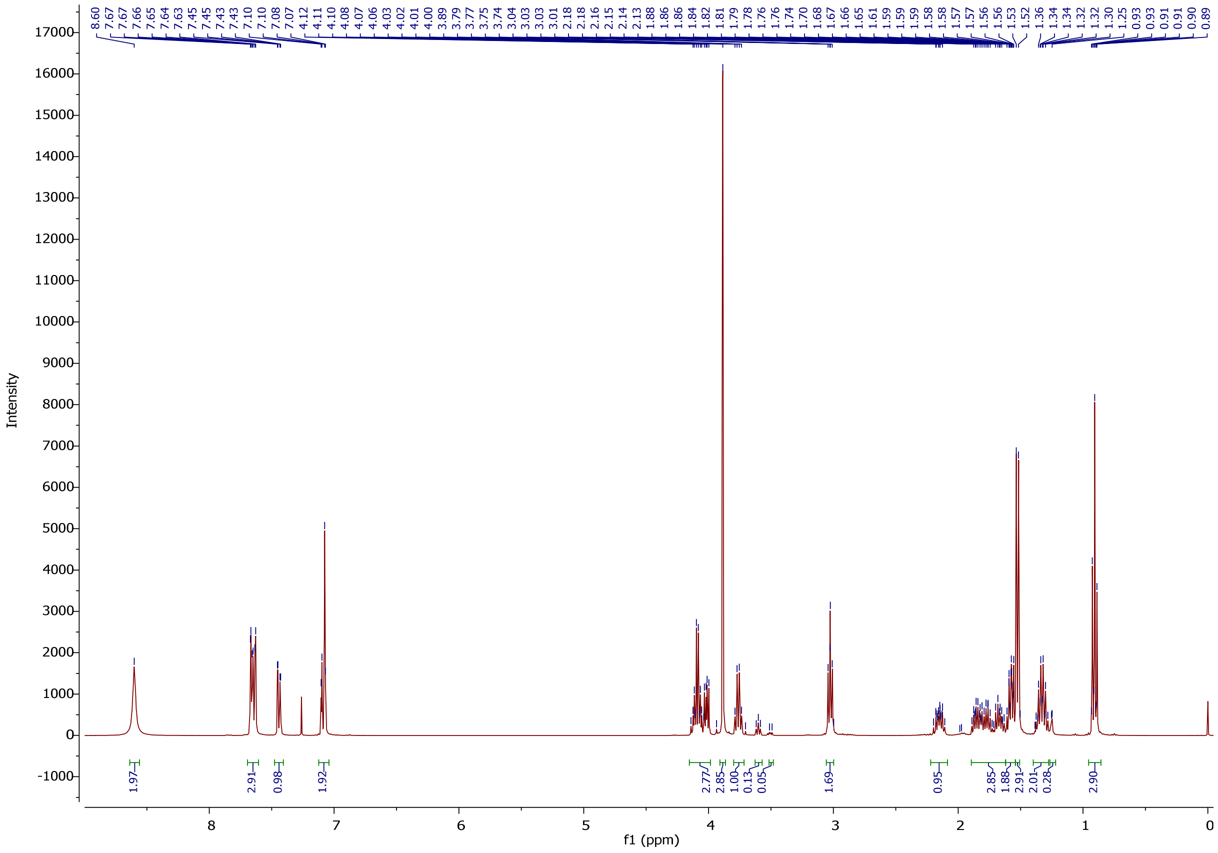

**Figure S7.** $^{1}H$ NMR spectra of L-proline butyl ester naproxenate.

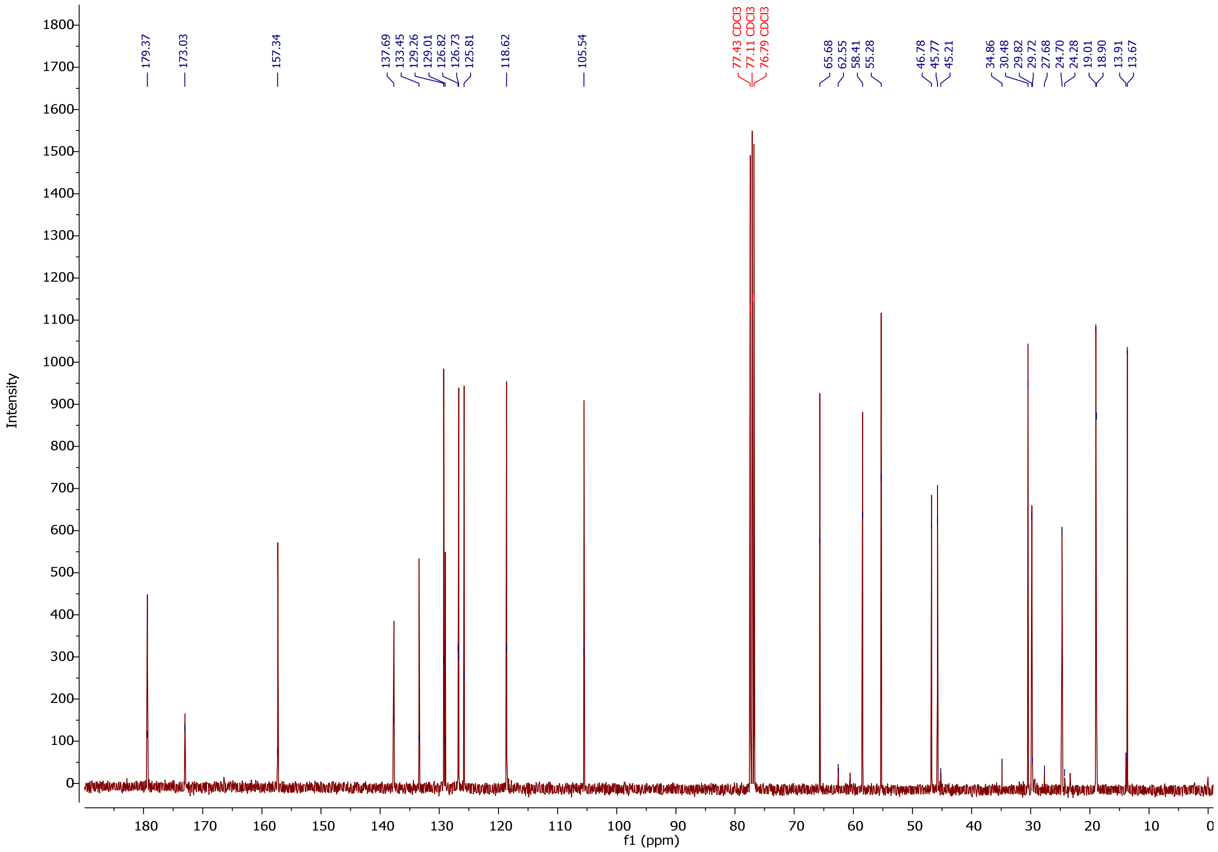


**Figure S8.** $^{13}$C NMR spectra of L-proline butyl ester naproxenate.

# The ATR-FTIR spectra of [ProOR][NAP]

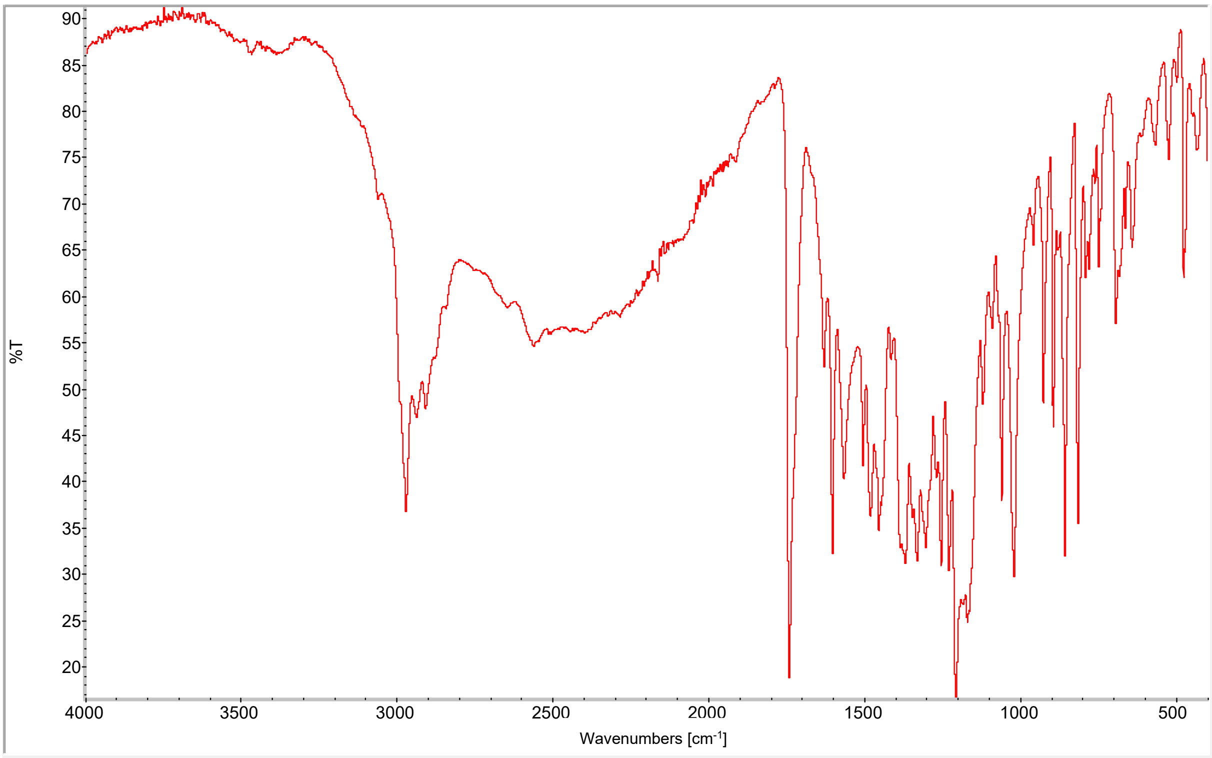


**Figure S9.** ATR-FTIR spectra of L-proline ethyl ester naproxenate.

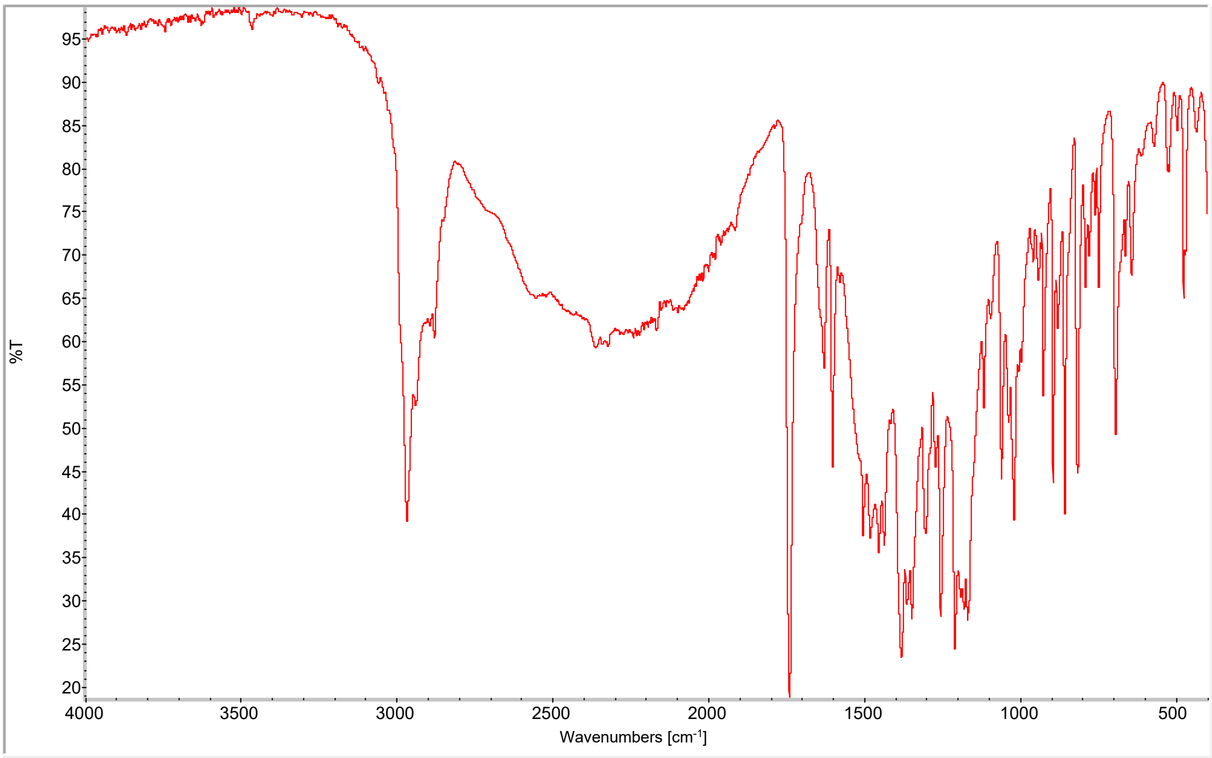


**Figure S10.** ATR-FTIR spectra of L-proline propyl ester naproxenate.

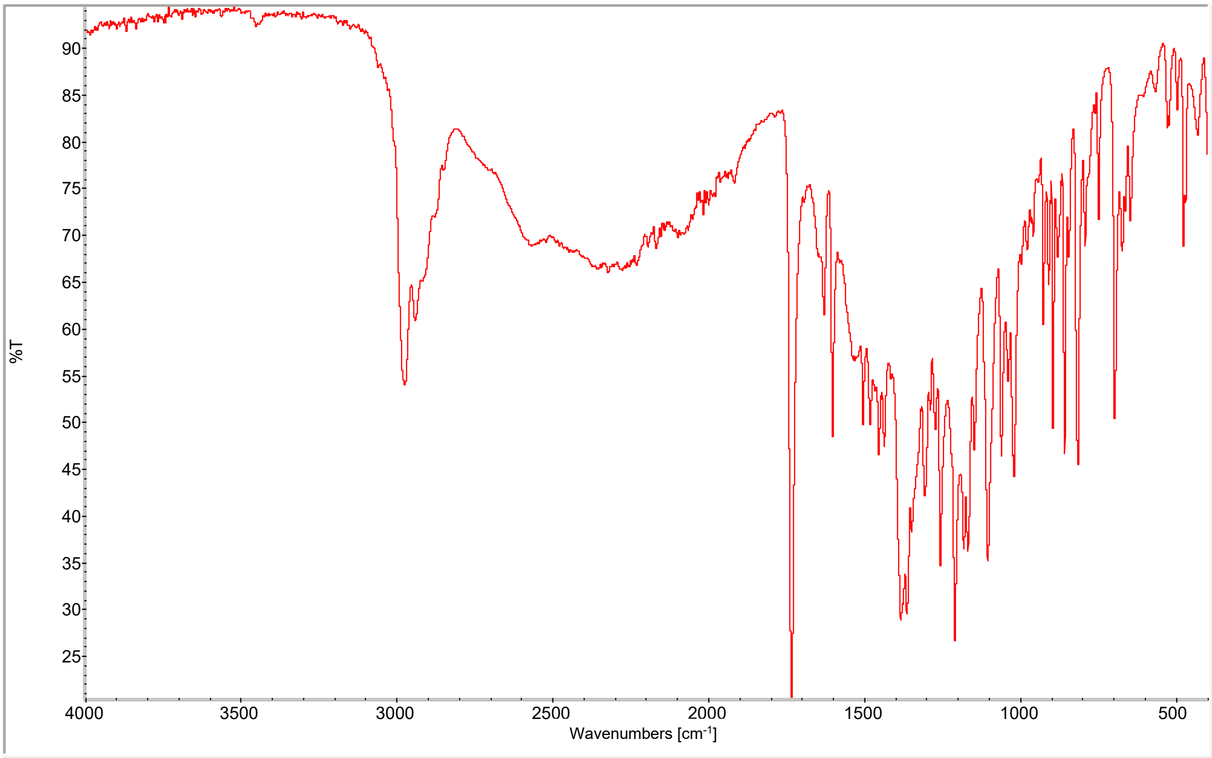


**Figure S11.** ATR-FTIR spectra of L-proline isopropyl ester naproxenate.

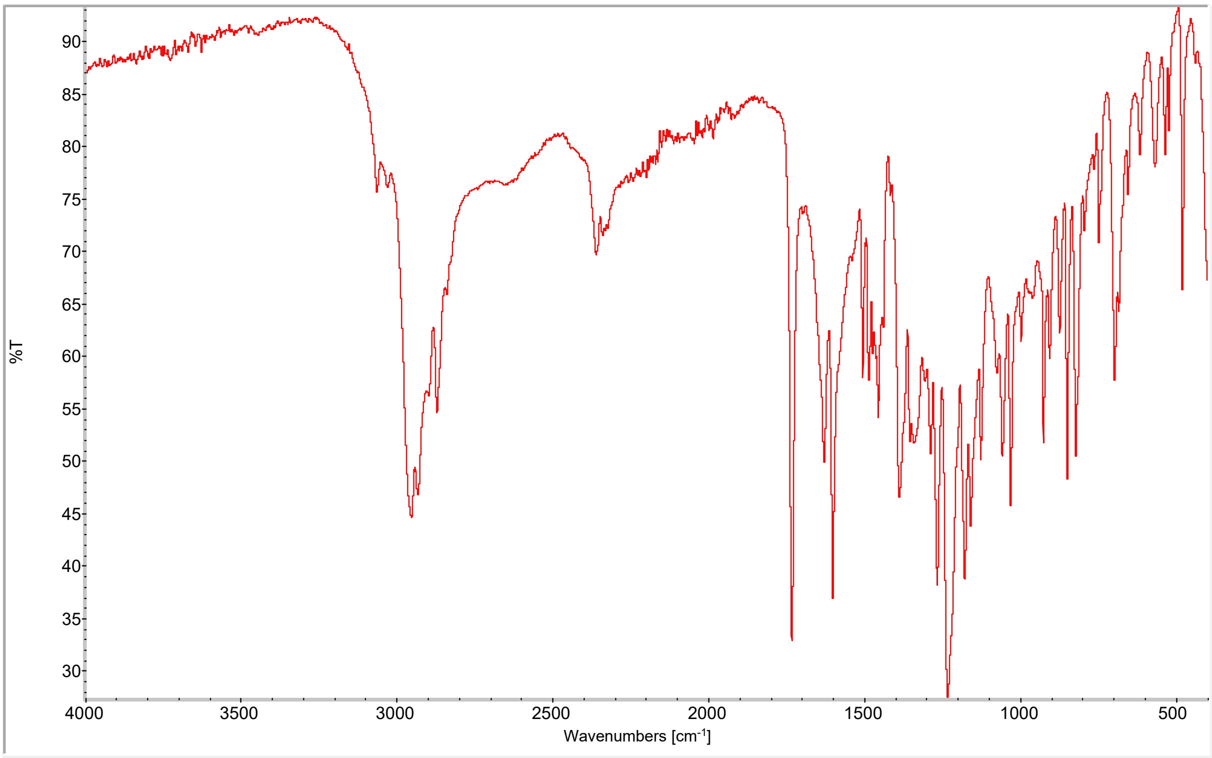


**Figure S12.** ATR-FTIR spectra of L-proline butyl ester naproxenate.

# The DSC curves of [ProOR][NAP]

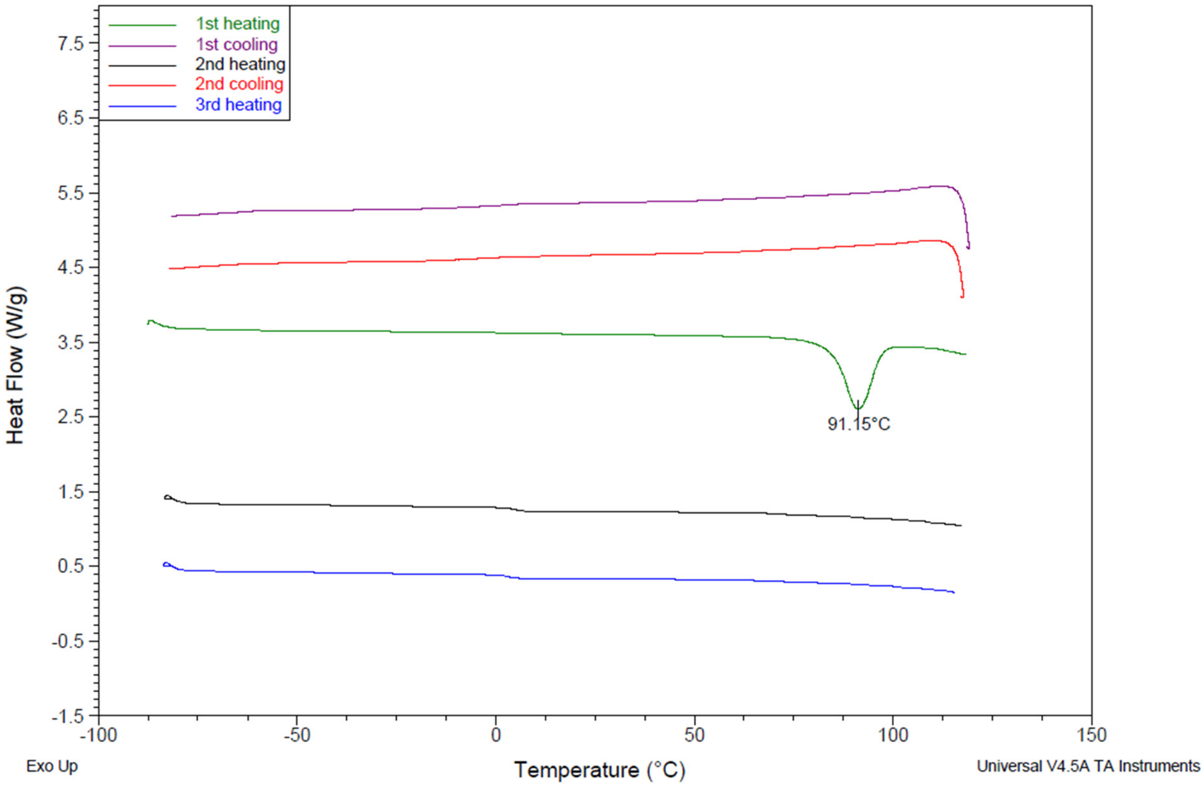


**Figure S13.** The DSC curves of L-proline ethyl ester naproxenate.

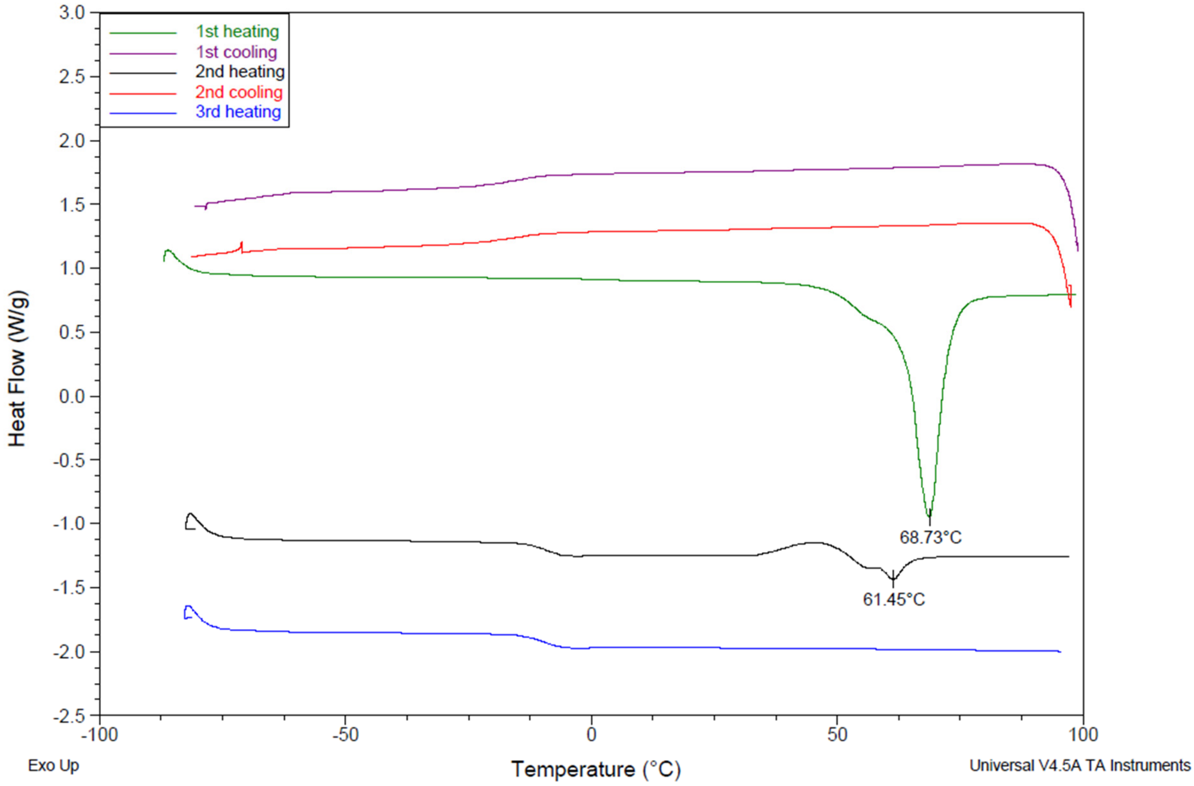


**Figure S14.** The DSC curves of L-proline propyl ester naproxenate.

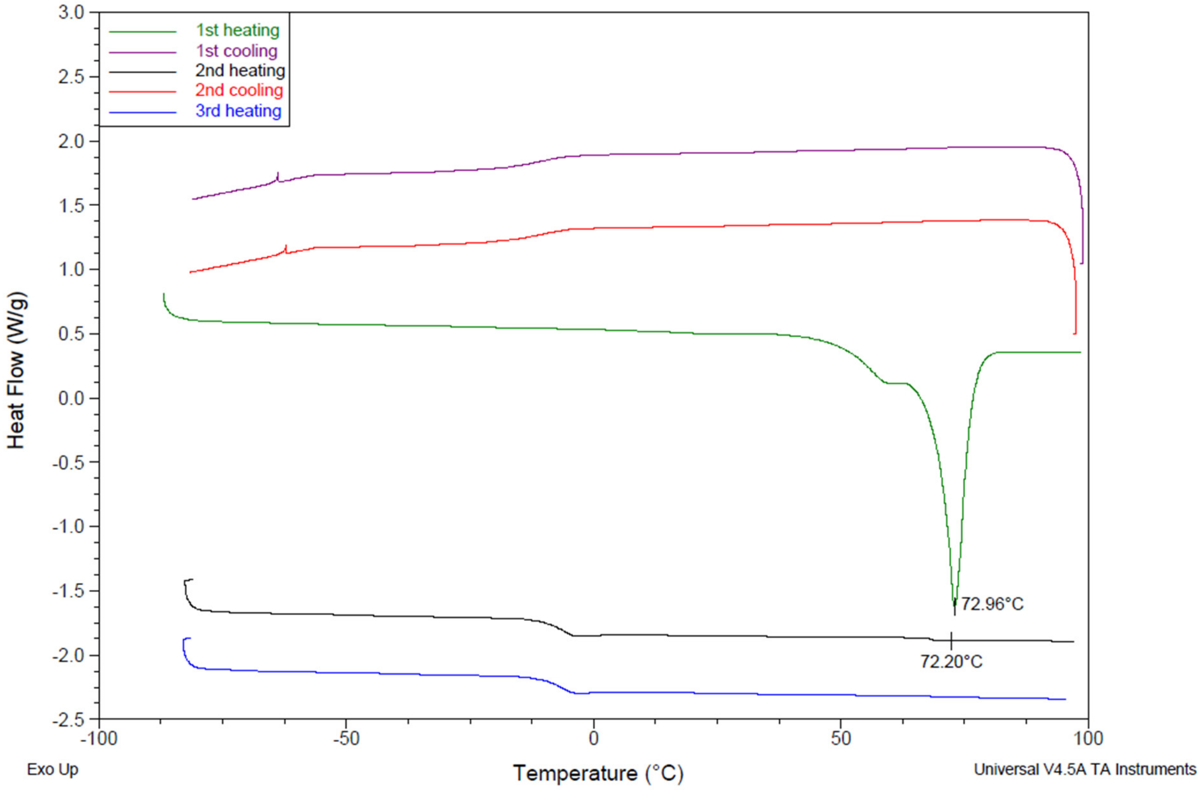


**Figure S15.** The DSC curves of L-proline isopropyl ester naproxenate.

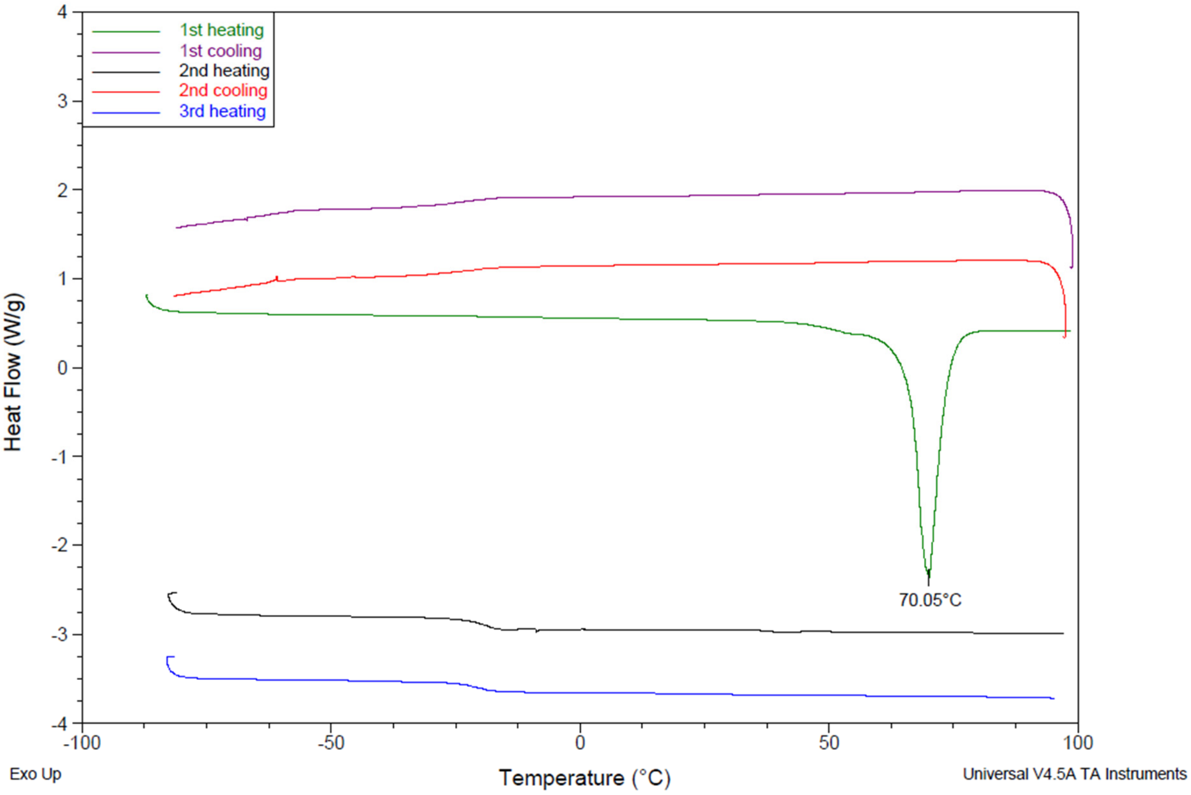


**Figure S16**. The DSC curves of L-proline butyl ester naproxenate.

## The TG curves of [ProOR][NAP]

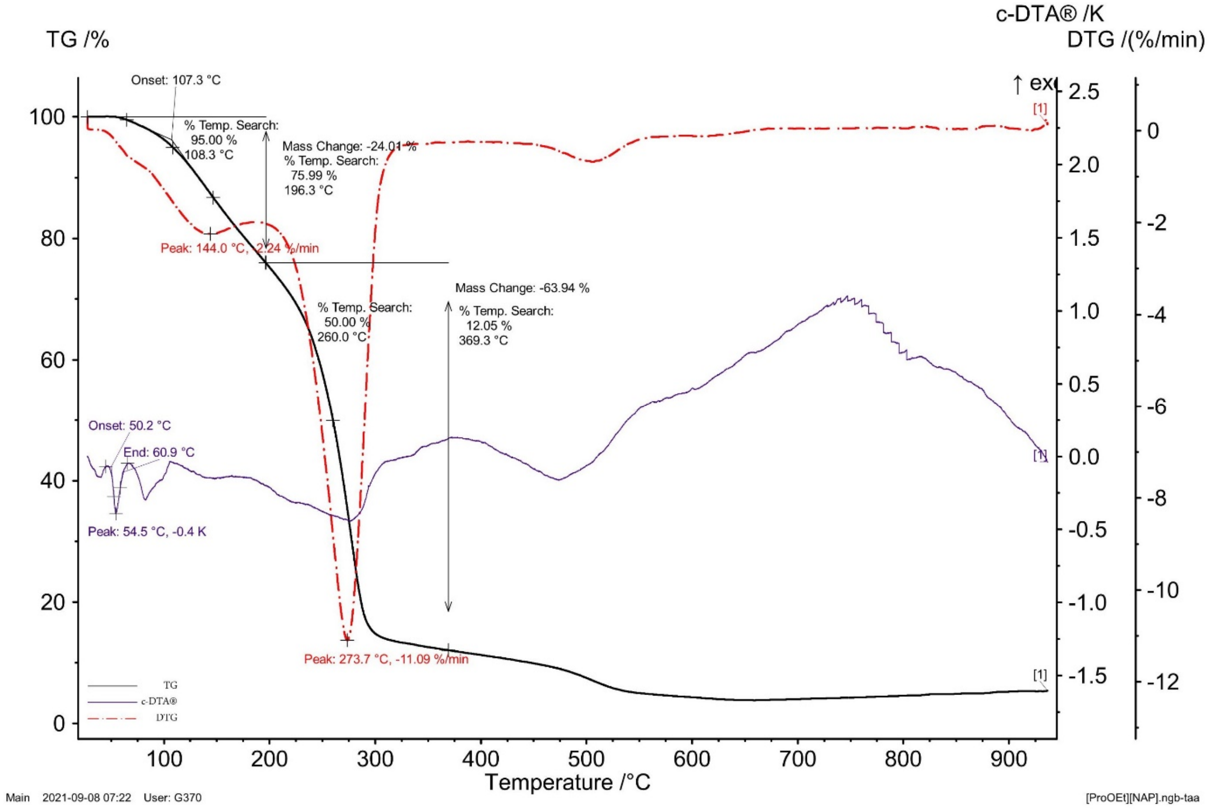


**Figure S17.** The TG, DTG and c-DTA curves of L-proline ethyl ester naproxenate.

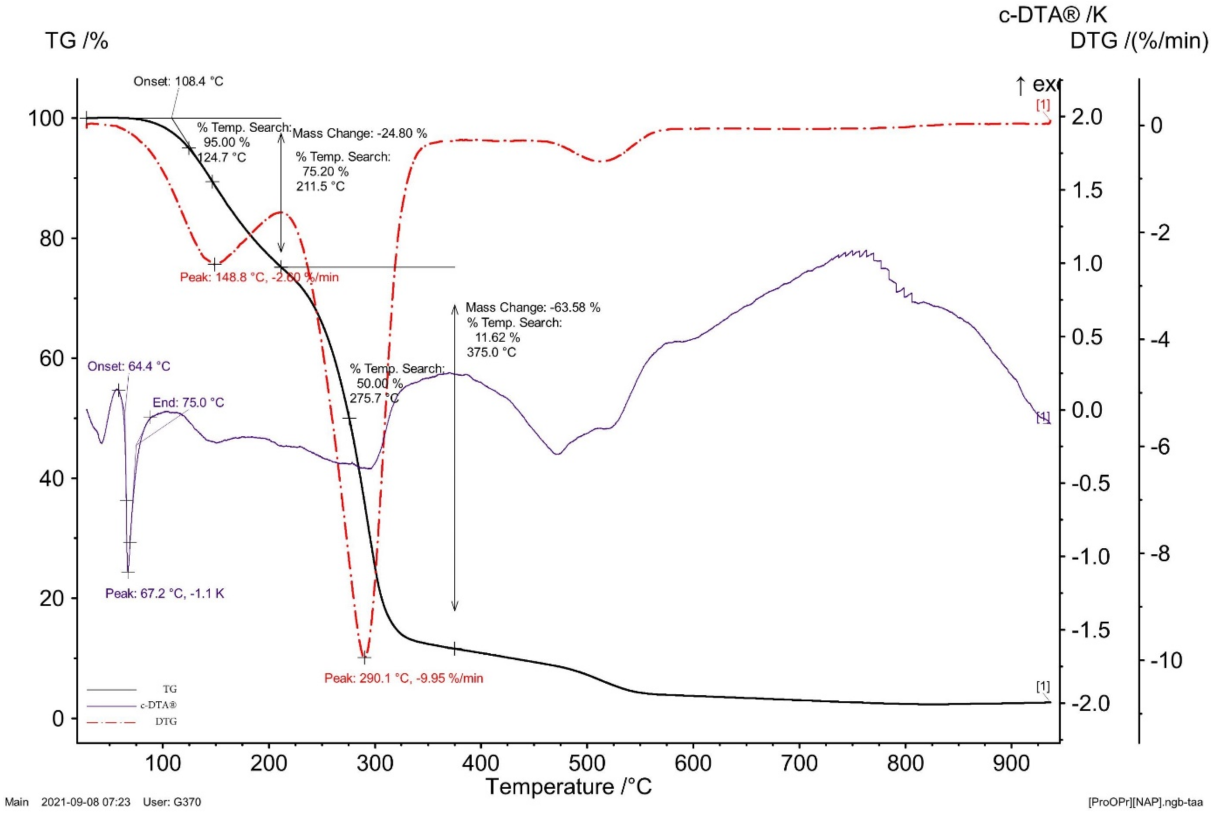


**Figure S18.** The TG, DTG and c-DTA curves of L-proline propyl ester naproxenate.

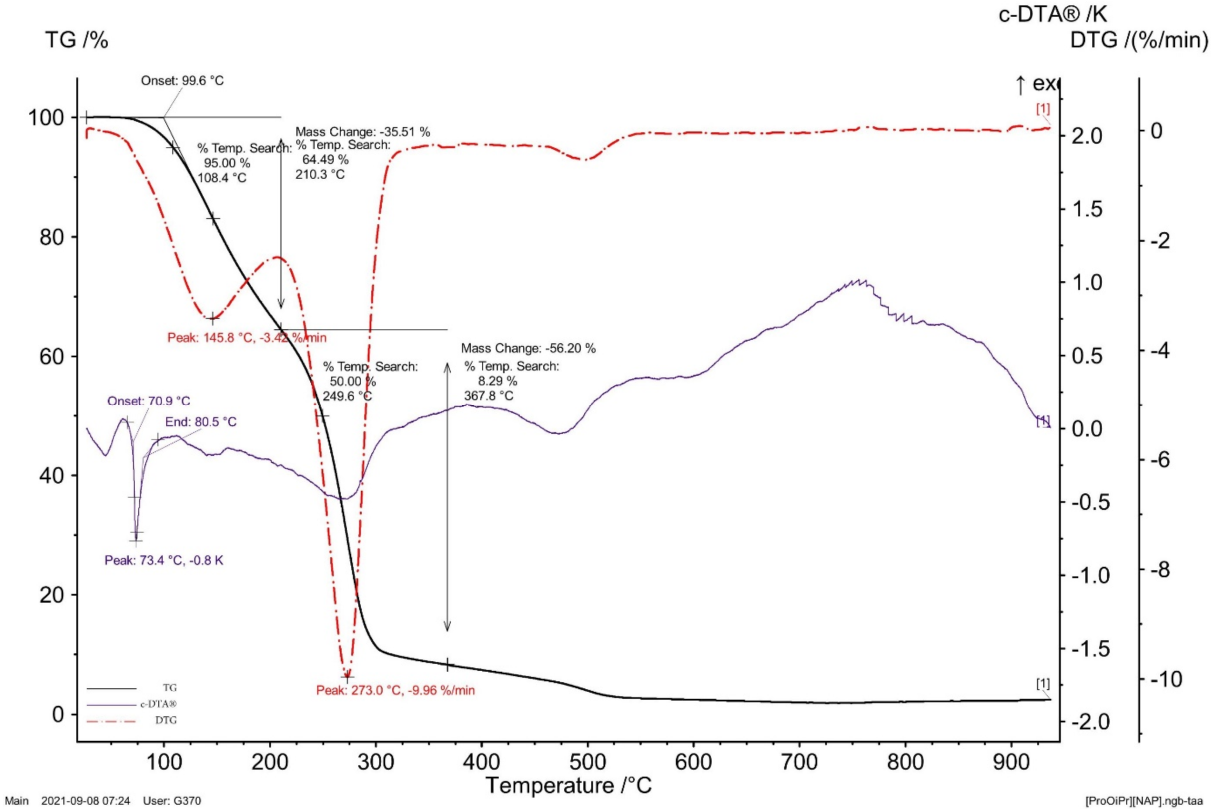


**Figure S17.** The TG, DTG and c-DTA curves of L-proline isopropyl ester naproxenate.

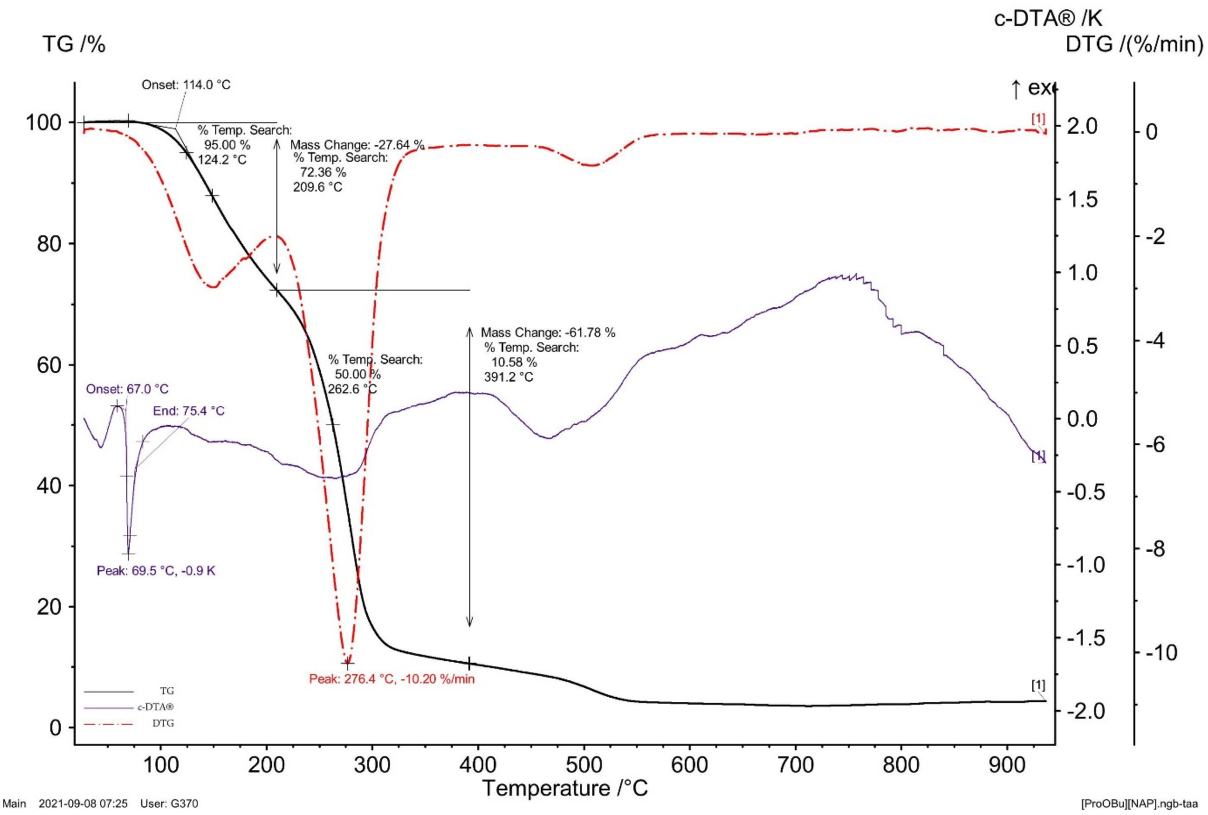


**Figure S20.** The TG, DTG and c-DTA curves of L-proline butyl ester naproxenate.

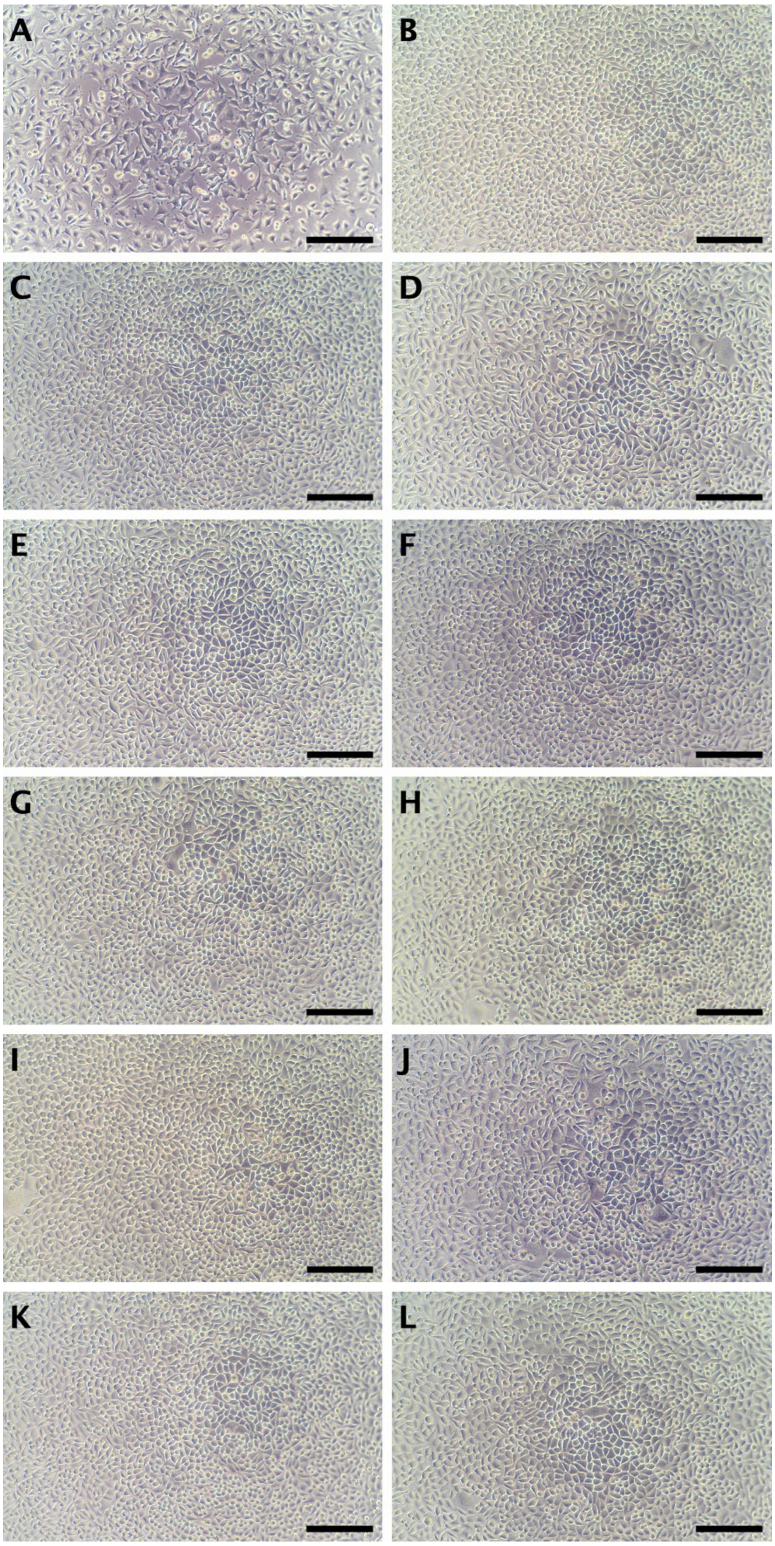


**Figure S18.** Representative micrographs of L929 cells. **A**) 24 hours after seeding. **B**) After further incubation for 24 hours with vehicle. Panels C–L present row-wise pairs of L929 cells incubated for 24 hours with the highest dose (right) and 3-fold dilution (left) of tested compounds. **C), D)**: [NAP]; **E**), **F**): [ProOEt][NAP]; **G**), **H**): [ProOPr][NAP]; **I**), **J**): [ProOiPr][NAP]; **K**), **L**): [ProOBu][NAP] Scale bar represents 200 μm.

# Method Validation

**Linearity**

Linearity was determined on solutions of naproxen and its derivatives working standards within the $1.6 \cdot 10^{-5}$ - 0.6 mmol/l range. Solutions were made by dissolving naproxen or its derivatives in acetonitrile-water (50:50 v/v) and run in triplicate. The data obtained from the linearity experiments were subjected to linear regression analysis, with the concentration of injected standard being plotted against the peak areas obtained.

The linear regression equation was $y = 1 \cdot 10^8$ x +684402 with a coefficient of determination ($r^2$) of 0.9973, where y = peak area and x = concentration of the solution. The coefficients of variation (CV) of the peak areas obtained ranged between 0.06–0.24%, while the standard errors of the slope and coefficient intercept were 0.00077 and -0.00552, respectively.

**Sensitivity**

The detection limit and limit of quantitation were calculated according to these equations:

1. The detection limit (DL) may be expressed as:

$$\mathrm{DL} = \frac{3\sigma}{\mathrm{S}}$$

2. The quantitation limit (QL) may be expressed as:

$$\mathrm{QL} = \frac{10\sigma}{\mathrm{S}}$$

Where:
σ - the standard deviation of the regression line,
S - the slope of the calibration curve [64].

The detection limit (LD) was 2.31 mmol/l, while the quantitation limit (QL) was 7.71 mmol/l. Thus, the LOD and LOQ results obtained demonstrated that the developed method was adequately sensitive for determining naproxen and its derivatives [64].

**Accuracy**

Accuracy was evaluated by analyzing solutions with known quantities of naproxen. Triplicate determinations at three concentration levels were used. The mean recoveries of the assays were assessed for compliance with the International Conference on Harmonization (ICH) guidelines [64]. The results for the recovery of naproxen indicate that the developed method is accurate with mean recovery in line with the ICH guidelines for the validation of analytical procedures with a mean recovery of 100% ± 2.0%

**Precision**

According to ICH guidelines, the parameters of repeatability and intermediate precision were evaluated as indicators of precision [64]. Repeatability was determined by a six times injection of naproxen working solution, while intermediate precision was evaluated by injecting five replicate solutions of naproxen working standard over three consecutive days. A coefficient of variation (CV) of <1.5% of the replicate injections was used as an acceptance criterion for method precision.

In all the determinations, the CV was <1.5%, and thus the proposed method was found to be precise
